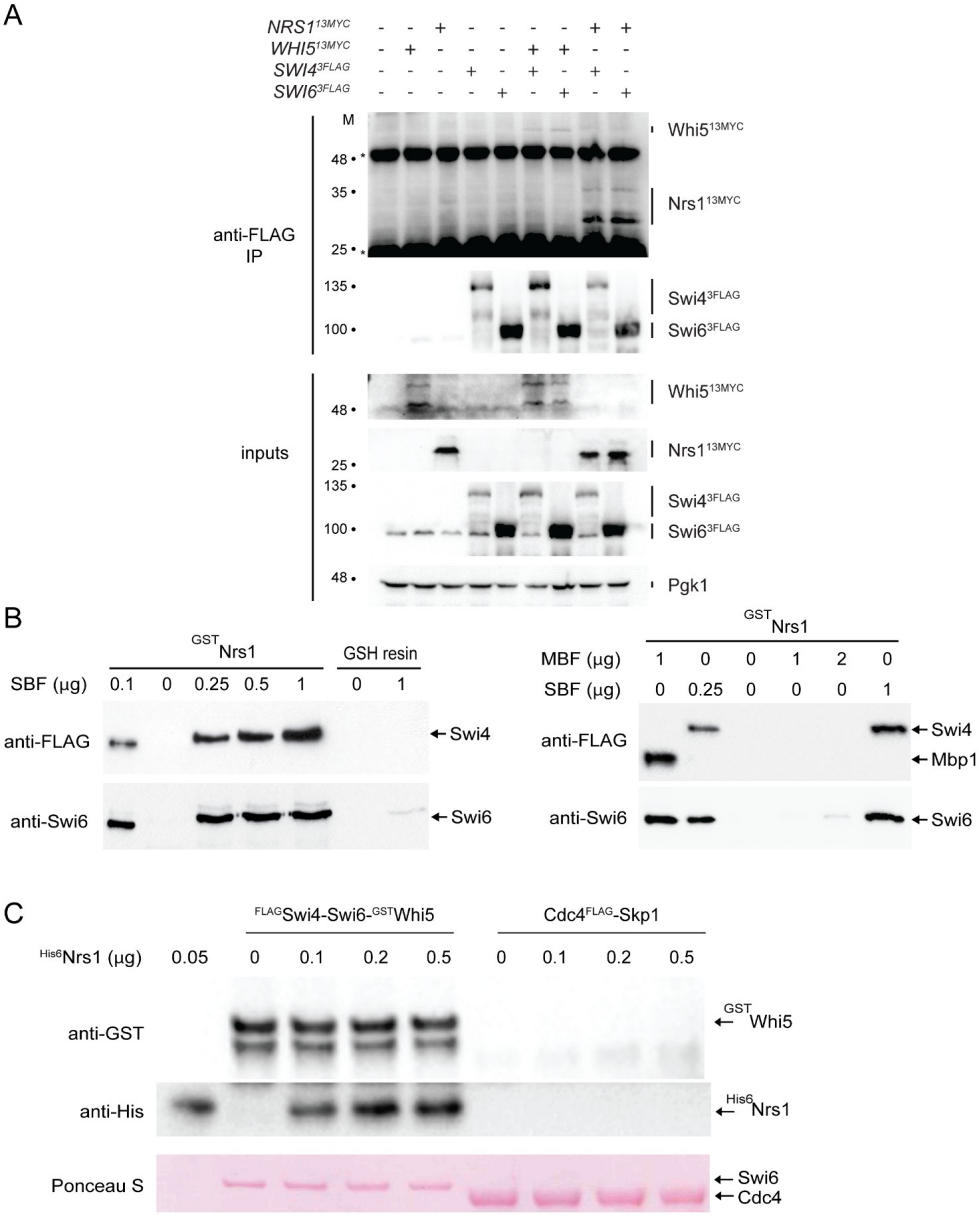# Correction: The microprotein Nrs1 rewires the G1/S transcriptional machinery during nitrogen limitation in budding yeast

**DOI:** 10.1371/journal.pbio.3001595

**Published:** 2022-03-08

**Authors:** The PLOS Biology Staff

## Notice of Republication

An incorrect version of Fig 5 was published in error. This article was republished on March 8, 2022 to correct for this error. Please download this article again to view the correct version.

**Fig 5:**