# Supplementary material for: The microprotein Nrs1 rewires the G1/S transcriptional machinery during nitrogen limitation in budding yeast
Source: PLoS Biol. 2022 Mar 3;20(3):e3001548. doi: 10.1371/journal.pbio.3001548 (PMC8893695; doi:10.1371/journal.pbio.3001548)
Supplement: S1 Fig — (A) Full Ylr053c/Nrs1 protein sequence is conserved across the Saccharomyces sensu stricto group of species. Sequence alignment showing the Ylr053c/Nrs1 protein sequence in S. cerevisiae (top), aligned with sequences of orthologs in S. bayanus (c672-g32.1), S. castellii (656.13d), S. paradoxus, and S. mikatae from top to bottom. Ylr053c/Nrs1 orthologs were not predicted in S. paradoxus or S. mikatae because of sharp length cutoffs in ORF prediction algorithms (the ORFs would span only 108 and 75 residues in S. paradoxus and S. mikatae, respectively). The lack of an obvious TATA box could also explain why no protein was predicted in S. paradoxus. Neighboring upstream and downstream genes both show high similarity to YLR053c neighbors in S. cerevisiae. The YLR053c/NRS1 sequence also aligns in S. kudriavzevii (not shown). (B) Kinetics of Nrs1 expression upon nitrogen starvation. sN&B images of untagged WT and NRS1-GFP log-phase cells (OD = 0.4 to 0.7), following 22 hours growth in YNB Pro medium from 1/5,000 dilution (left) and 7 hours growth from 1/100 dilution (right) from saturated precultures. Arrows indicate representative Nrs1 signal beyond autofluorescence at 22 hours. (C) Prolonged exposure to rapamycin results in accumulation of a faster migrating form of Nrs1. Rapamycin was added to log-phase cultures of NRS113MYC cells, aliquots were removed at indicated time intervals, and immunoprecipitates were analyzed by anti-MYC immunoblot. A raw image of the original immunoblot is provided in the S1 Raw Images. (D) Nuclear localization of Nrs1 upon rapamycin treatment is not a consequence of the particular GFPmut3 fluorophore. Confocal microscopy image of NRS1WT-GFP cells grown in SC + 2% glucose medium, either untreated or treated with 200 ng/mL rapamycin for 2 hours. (E) sN&B images of untagged WT cells and NRS1-GFP cells grown to log-phase in SC + 2% glucose and plated on SC + 2% glucose agar pads containing either 0.5 M NaCl or 1 mM H2O2 and imaged over a 2-hour t [file pbio.3001548.s001.pdf]

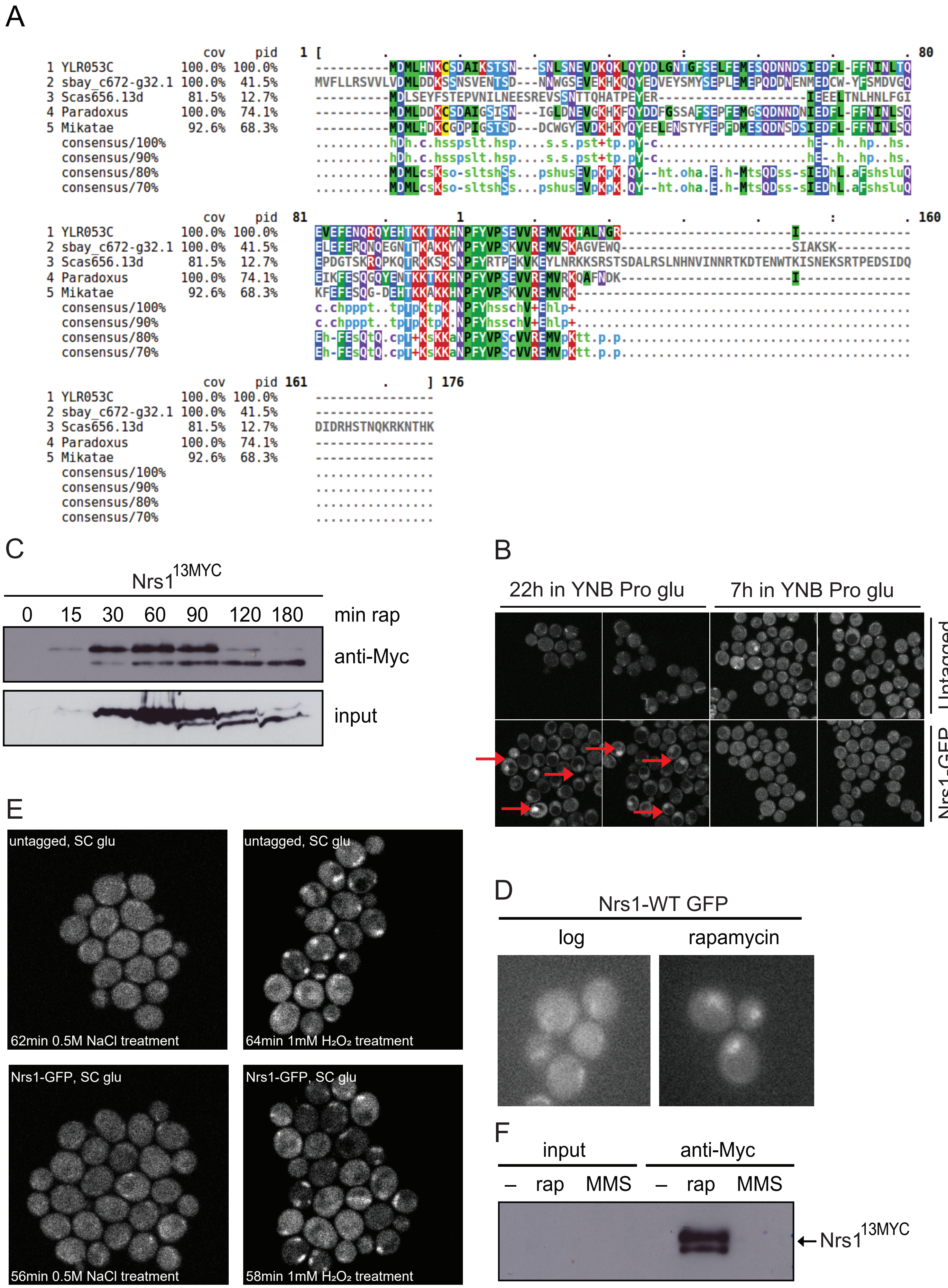

Tollis *et al.* Supp. Figure S1
