# Supplementary figures and images for: The microprotein Nrs1 rewires the G1/S transcriptional machinery during nitrogen limitation in budding yeast
Source: PLoS Biol. 2022 Mar 3;20(3):e3001548. doi: 10.1371/journal.pbio.3001548 (PMC8893695; doi:10.1371/journal.pbio.3001548)

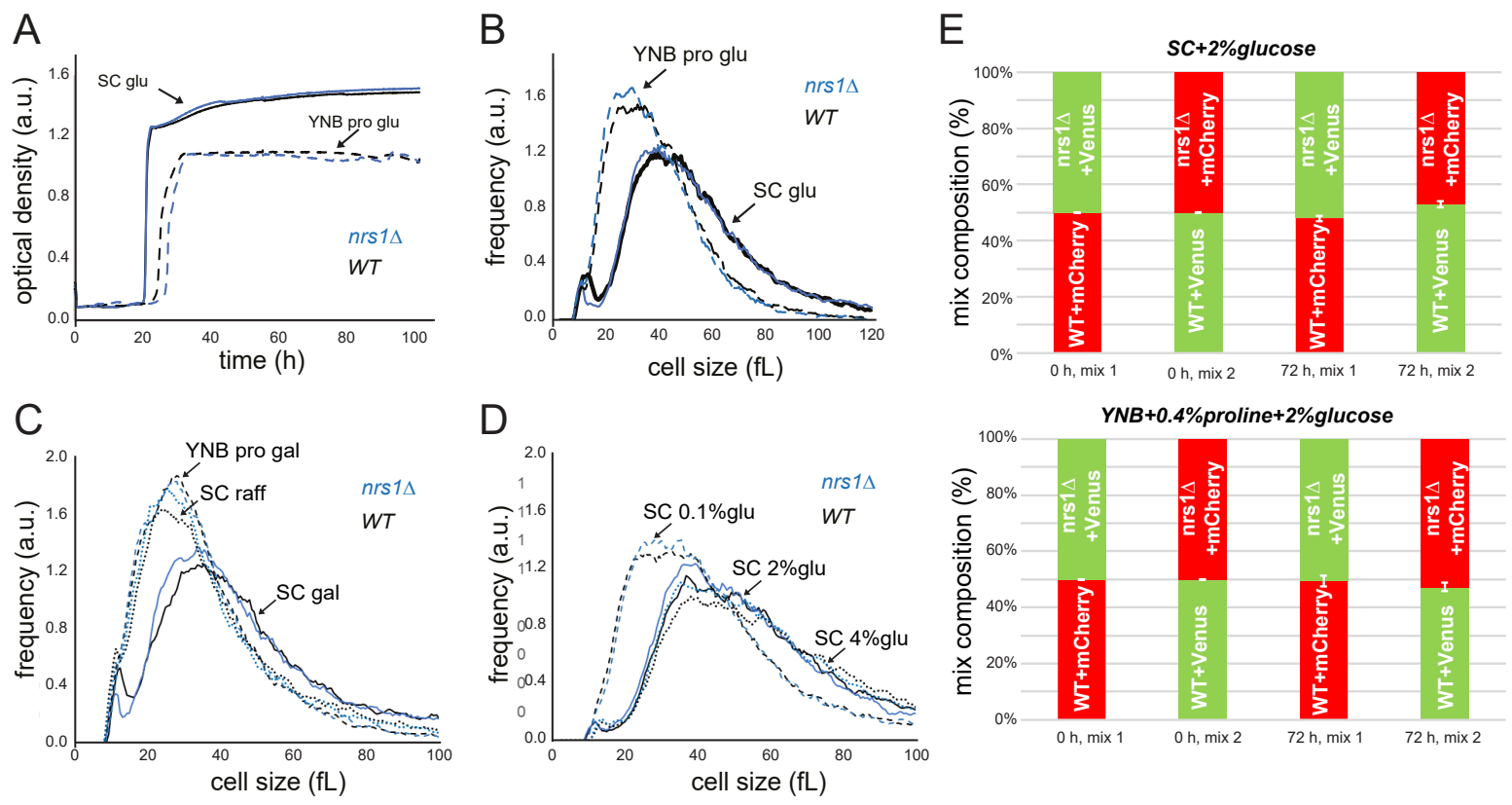

Supplement: S2 Fig — (A) Deletion of NRS1 does not affect growth. Optical density (vertical axis) of WT (black) and nrs1Δ (blue) strains grown in SC + 2% glucose (solid lines) or nitrogen-limited (YNB+Pro, dashed lines) medium as a function of time (horizontal axis). (B–D) Deletion of NRS1 does not affect cell size. Cell size distributions of WT (black) and nrs1Δ (blue) strains grown in SC + 2% glucose (B, solid lines), nitrogen-limited (B, YNB+Pro, dashed lines), SC + 2% galactose (C, solid lines), SC + 2% raffinose (C, dotted lines), YNB + 0.4% proline + 2% galactose (C, YNB pro gal, dashed lines), SC + 2% glucose (D, solid lines), SC + 4% glucose (D, dotted lines) and SC + 0.1% glucose (D, dashed lines). (E) Deletion of NRS1 does not affect competitive fitness in SC + 2% glucose and nitrogen limited (YNB+Pro) medium during growth to stationary phase. Bar charts representation of the composition of 2 mixes of competing strains (Mix1: WT transformed with mCherry plasmid (red) and nrs1Δ transformed with Venus plasmid (green); Mix2: WT with Venus plasmid (green), and nrs1Δ with mCherry plasmid (red)) as a function of time from inoculation. The percentage of each strain within the mixes shown is derived from 3 replicate cultures from the same original mixes (see S1 Text Methods). Error bars show the standard error on the mean. All numerical values underlying this figure may be found in S2 Data. NRS1, Nitrogen-Responsive Start regulator 1; WT, wild-type. (PDF) [file pbio.3001548.s002.pdf]

A

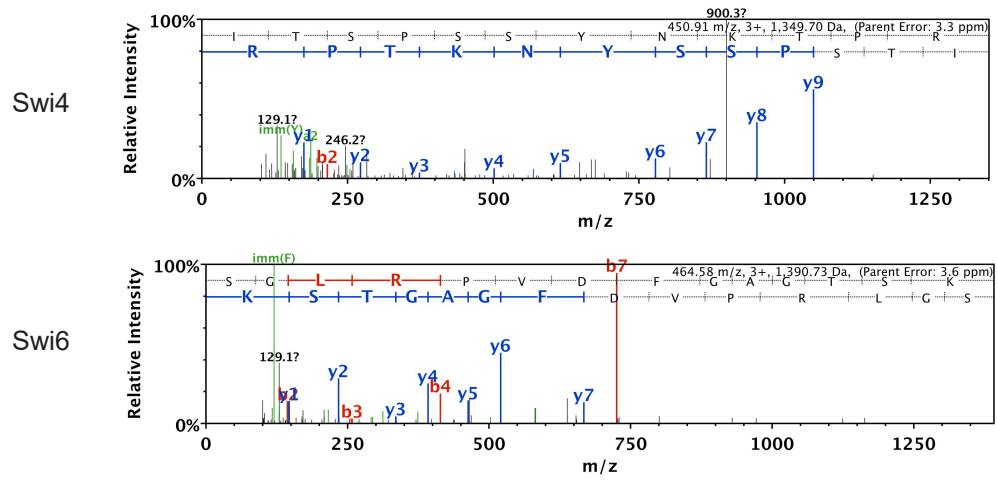

B

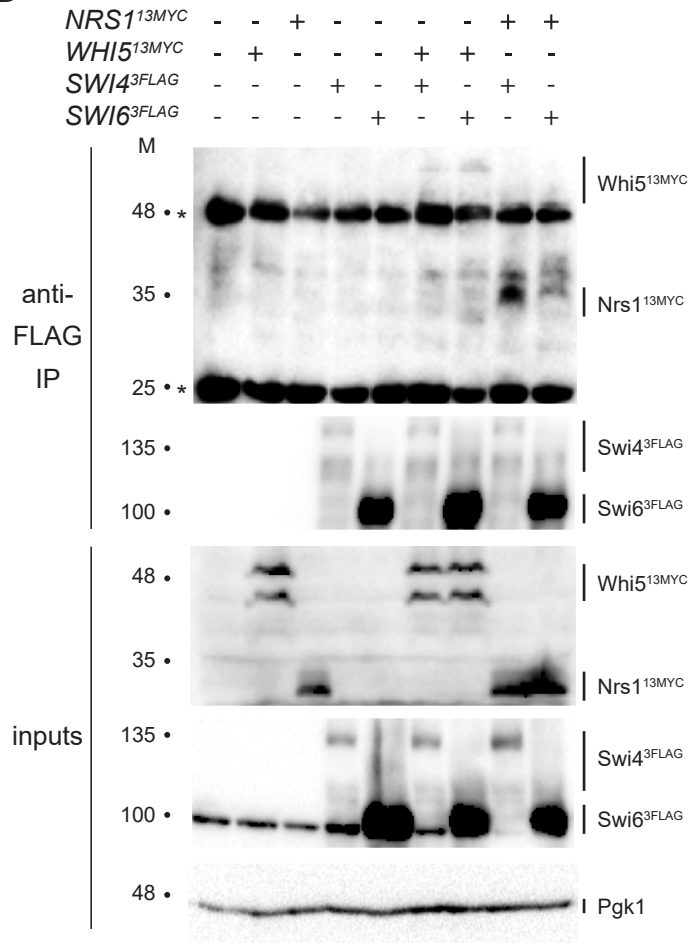

C

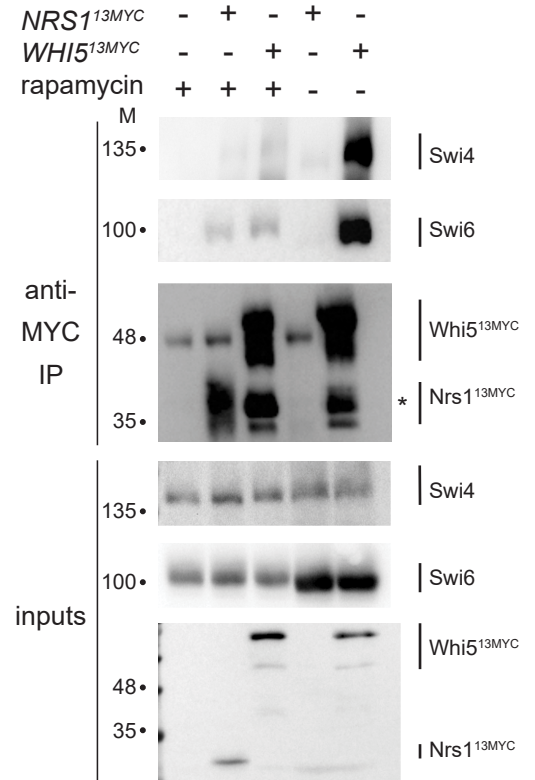

Supplement: S3 Fig — (A) Example Swi4 and Swi6 peptide spectra detected in Nrs1 immunoprecipitates. The first of the 5 peptides identified for Swi4 (ITSPSSYNKTPR) and Swi6 (SGLRPVDFGAGTSK) are shown on top and bottom, respectively. Data were processed with Scaffold software. (B) Replicate experiment for interactions detection with endogenous level of tagged proteins (Fig 5A). Swi43FLAG or Swi63FLAG complexes were immunoprecipitated from the indicated strains grown in the presence of 200 nM rapamycin for 3 hours and interacting proteins assessed by immunoblot with the indicated antibodies. Co-immunoprecipitation of Whi513MYC with Swi43FLAG and Swi63FLAG served as a positive control. Mr markers (M) are indicated for each blot. Pgk1 served as a loading control. Asterisk indicates IgG heavy and light chains. (C) Detection of endogenous untagged Swi4 and Swi6 in Nrs113MYC immunoprecipitates. Nrs113MYC or Whi513MYC complexes were immunoprecipitated from cultures of the indicated strains that were either untreated or treated with 200 nM rapamycin for 3 hours. Interacting proteins assessed by immunoblot with the indicated antibodies. Co-immunoprecipitation of Swi4 and Swi6 with Whi513MYC served as a positive control. Double asterisk indicates Whi513MYC degradation product that migrated at a similar size as Nrs113MYC. Mr markers (M) are indicated for each blot. NRS1, Nitrogen-Responsive Start regulator 1; SBF, SCB-binding factor. (PDF) [file pbio.3001548.s003.pdf]

A

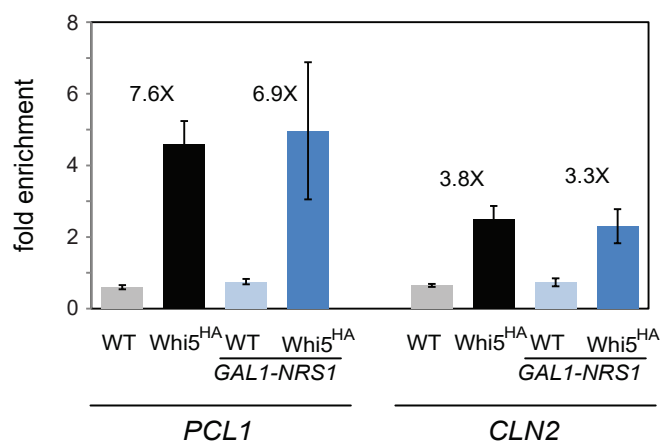

B

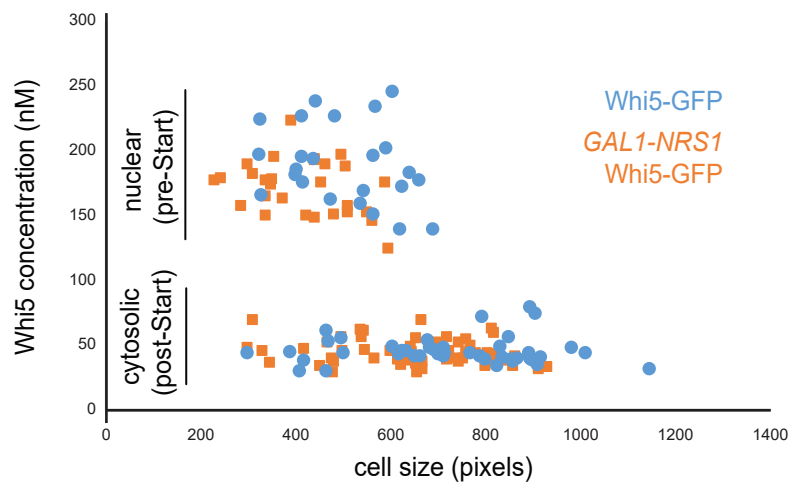

C

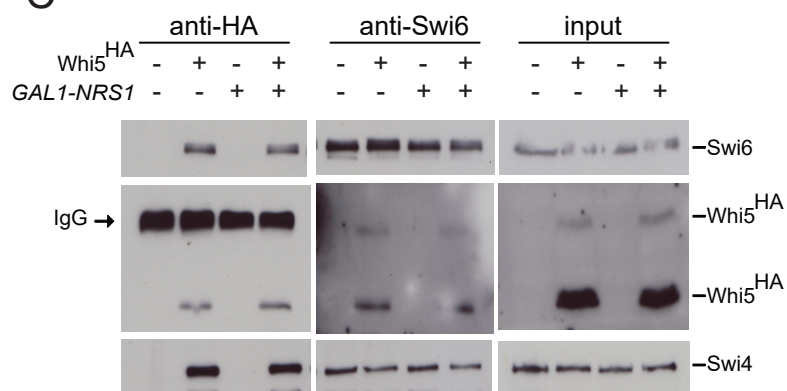

D

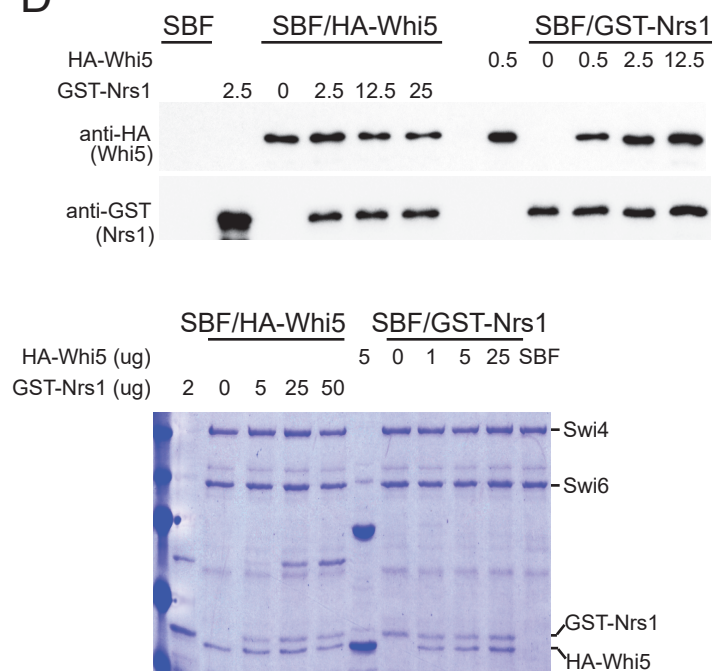

Supplement: S4 Fig — (A) NRS1 overexpression does not inhibit Whi5 association with G1/S promoter DNA. WT or WHI5HA strains carrying empty vector or a GAL1-NRS1 plasmid were grown in SC + 2% raffinose medium and induced with 2% galactose for 6 hours prior to crosslinking. Anti-HA ChIPs were assessed for the presence of CLN2 and PCL1 promoter DNA by quantitative RT-PCR. Bars indicate the mean fold-enrichment across 2 replicates, and error bars show the standard error on the mean. (B) NRS1 overexpression does not affect Whi5 protein levels. Whi5-GFP absolute concentration in single WT (blue dots) and GAL1-NRS1 (orange dots) cells first grown in SC + 2% raffinose then induced with 2% galactose for 6 hours prior to sN&B microscopy. Nuclear Whi5-GFP levels in pre-Start cells and cell-averaged levels in post-Start cells where Whi5 has been exported from the nucleus are shown. All numerical values underlying panels A and B may be found in S4 Data. (C) NRS1 overexpression does not inhibit Whi5 association with SBF. The indicated Whi5HA immunoprecipitates from strains induced with galactose for 6 hours were probed for Whi5HA, Swi4, or Swi6 by immunoblot. (D) Nrs1 does not compete with Whi5 for binding to SBF in vitro. The indicated amounts of recombinant HAWhi5 or GSTNrs1 was titrated into preformed FLAGSwi4-Swi6-GSTNrs1 or FLAGSwi4-Swi6-HAWhi5 complexes immobilized on anti-FLAG resin, respectively. Bound proteins were resolved by SDS-PAGE then immunoblotted (top) or stained with Coomassie Brilliant Blue (bottom). Note that added soluble GSTNrs1 or HAWhi5 saturated the respective SBF-HAWhi5 and SBF-GSTNrs1 complexes at the lowest input concentrations. Raw image of the original immunoblots used to made panels C and D are provided in S1 Raw Images. ChIP, chromatin immunoprecipitation; NRS1, Nitrogen-Responsive Start regulator 1; SBF, SCB-binding factor; sN&B, scanning Number and Brightness; WT, wild-type. (PDF) [file pbio.3001548.s004.pdf]

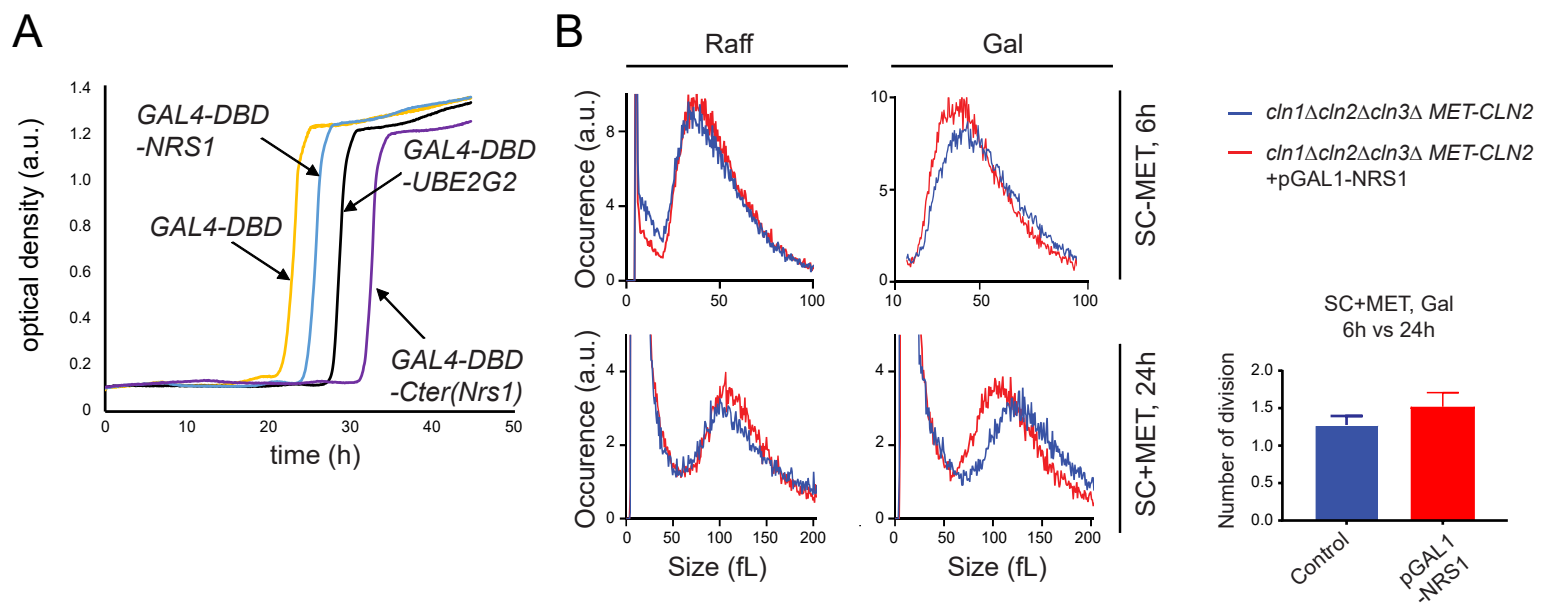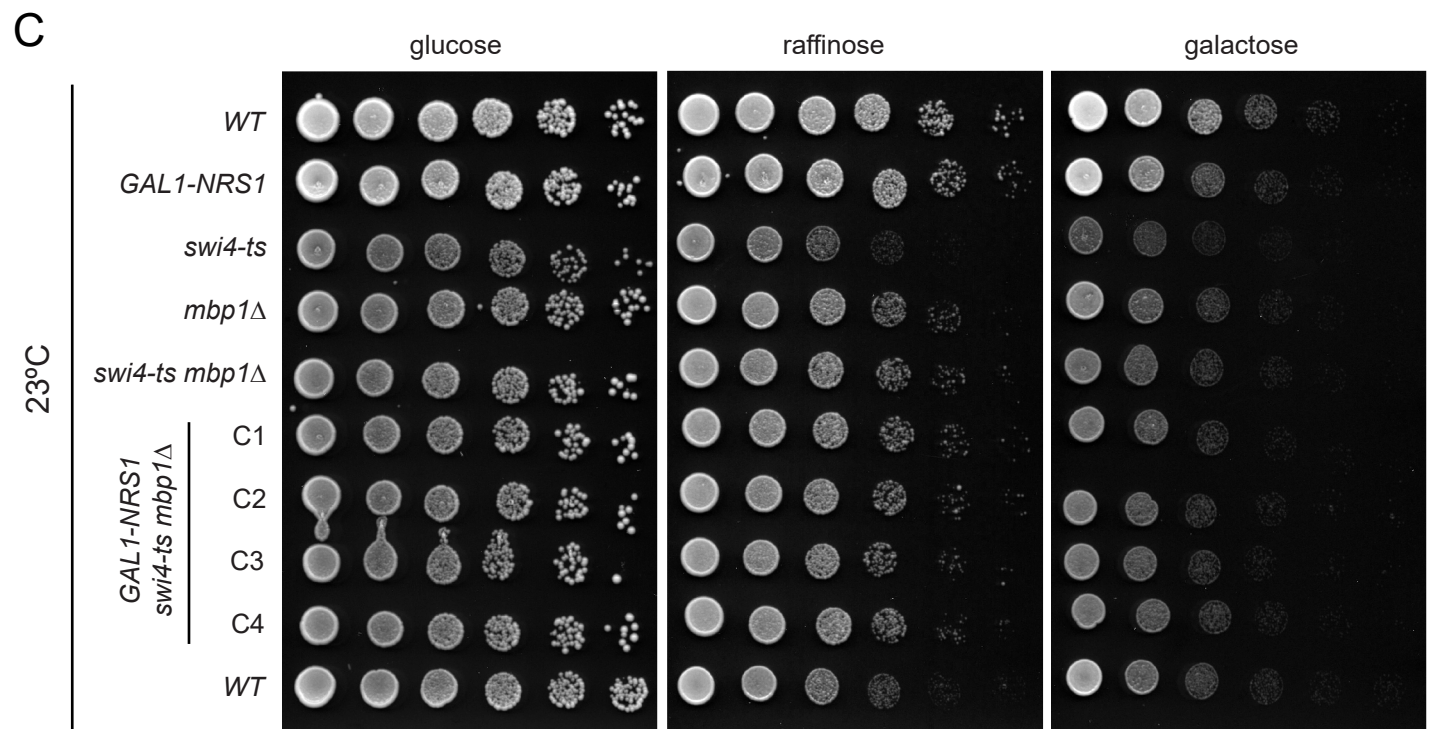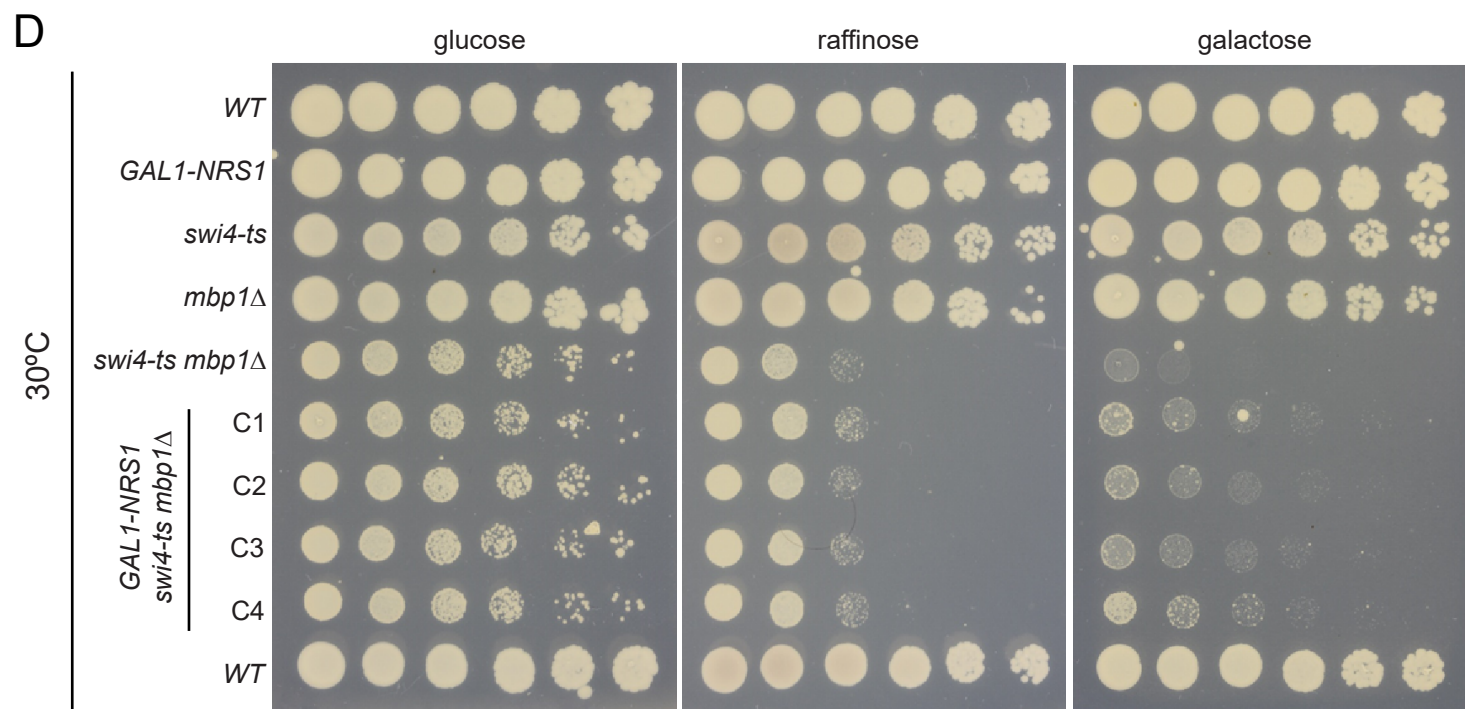

Supplement: S5 Fig — (A) Control growth curves for transactivation assays. Reporter strains transformed with plasmids expressing either GAL4DBD alone, GAL4DBD-NRS1, GAL4DBD-UBE2G2, or GAL4DBD-NRS1Cter were grown in SD-Trp medium at 30°C. (B) NRS1 overexpression does not rescue a cln1Δcln2Δcln3Δ G1 phase arrest. Left: Cultures of cln1Δcln2Δcln3Δ MET-CLN2 and cln1Δcln2Δcln3Δ MET-CLN2 + strains grown to log-phase in SC-Met+2% raffinose, then reinoculated in either SC-Met+2% raffinose, SC-Met+2% galactose, SC+Met+2% raffinose or SC+Met+2% galactose for the indicated periods of time before determination of cell size distributions on a Beckman Z2 Coulter counter. Right: bar charts showing the average number of cell divisions for cln1Δcln2Δcln3Δ MET25-CLN2 and cln1Δcln2Δcln3Δ MET25-CLN2 + strains during the 18 hour interval between the 6 hours and 24 hours time points in SC+Met+2%galactose. Bar heights represent the average of 4 different clones (N = 4); error bars represent the standard deviation. (C) Room temperature growth controls for genetic interactions of NRS1 with SWI4 and MBP1. Serial 5-fold dilutions of WT NRS1 and nrs1::GAL1-NRS1 strains in WT (rows 1, 2, 10), swi4-ts (row 3), mbp1Δ (row 4), and mbp1Δswi4-ts (rows 5–9) backgrounds were spotted onto SC + 2% glucose, SC + 2% raffinose, and SC + 2% galactose medium and grown for 5 days at 23°C. C1-4 are 4 clones of mbp1Δ swi4-ts GAL1-NRS1. (D) Images of the same serial 5-fold dilutions of NRS1 and GAL1-NRS1 strains in WT, swi4-ts, mbp1Δ, and mbp1Δ swi4-ts backgrounds as in Fig 7B, spotted onto SC + 2% glucose, SC + 2% raffinose and SC + 2% galactose, but grown for an additional 2 days (i.e., 7 days total growth time at 30°C). C1 to C4 are 4 clones of mbp1Δ swi4-ts GAL1-NRS1. All numerical values underlying panels A and B may be found in S6 Data. NRS1, Nitrogen-Responsive Start regulator 1; WT, wild-type. (PDF) [file pbio.3001548.s005.pdf]

A

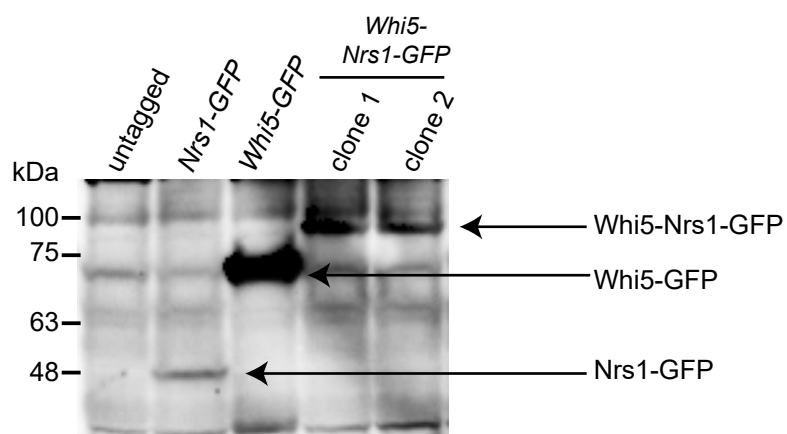

B

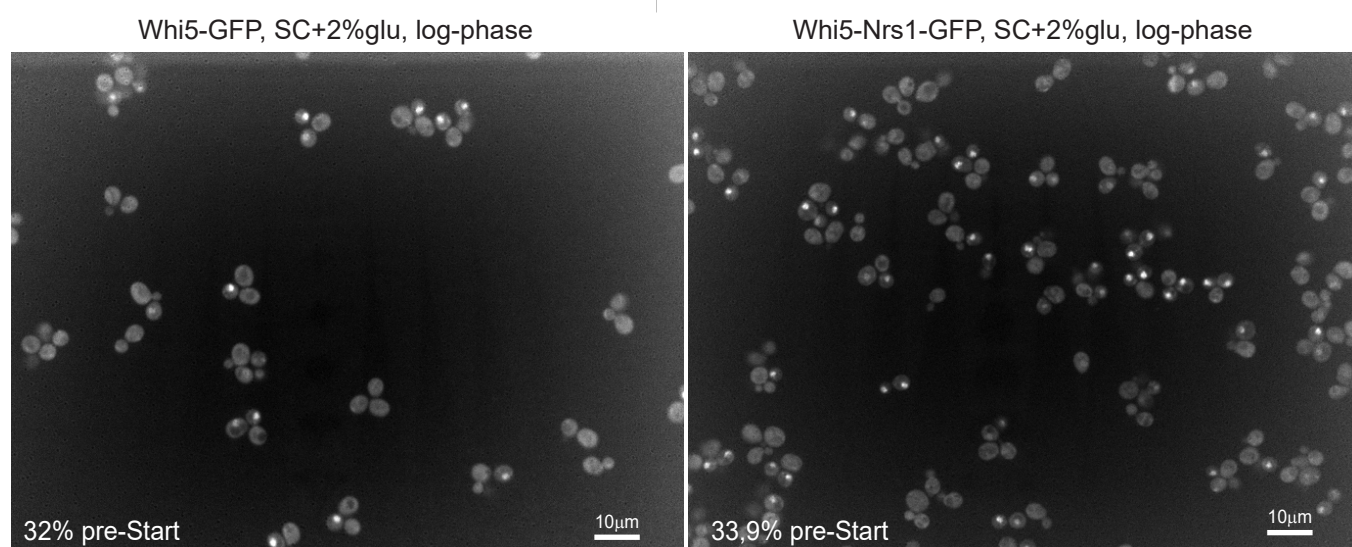

C

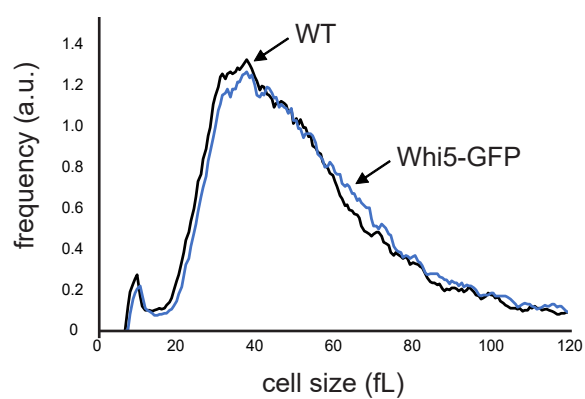

D

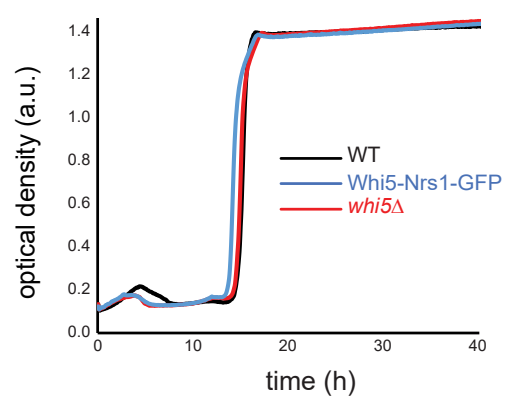

E

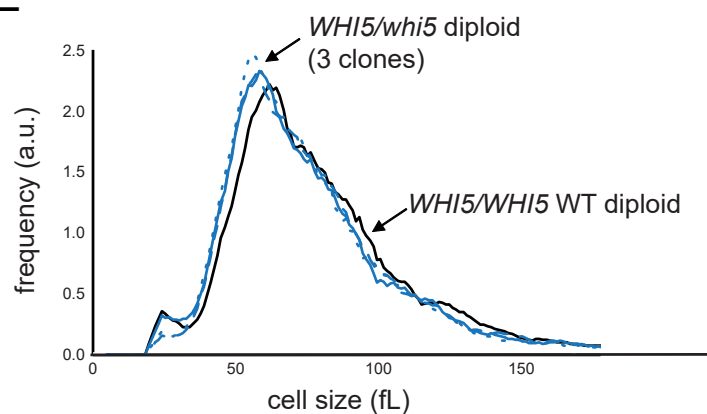

F

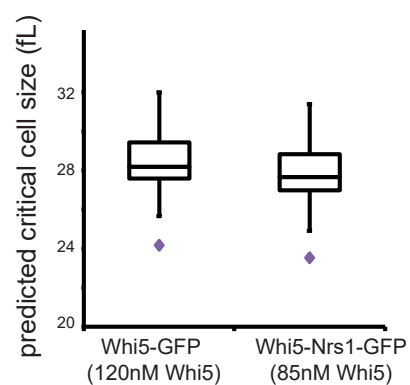

Supplement: S6 Fig — (A) The Whi5-Nrs1-GFP chimeric protein is produced in vivo and migrates at the expected size. WHI5-GFP, NRS1-GFP, and WHI5-NRS1-GFP strains were grown in nitrogen-limited (YNB+Pro) medium and extracts immunobloted with anti-GFP antibody. A raw image of the original immunoblot is provided in S1 Raw Images. (B) A carboxyl-terminal fusion of Nrs1 to Whi5 does not affect cell cycle distribution. High-content images of WHI5-GFP and WHI5-NRS1-GFP cells grown in SC + 2% glucose were acquired on an OPERA high-throughput confocal microscope (PerkinElmer) equipped with a 60× water objective. The same intensity scale was used for both panels. Scale bar is 10 μm. The fraction of pre-Start (G1) cells was obtained using a custom MATLAB script (see Methods). (C) Fusion of a GFP tag at the Whi5 carboxyl terminus does not affect cell size. Cell size distributions of untagged WT and WHI5-GFP cells grown in SC + 2% glucose were determined on a Beckman Z2 Coulter counter. (D) Growth curves of WT, whi5Δ, and WHI5-NRS1-GFP strains in SC + 2% glucose medium at 30°C. (E) WHI5 dosage has only minor effects on cell size. Cell size distributions of WT and WHI5/whi5 heterozygous diploid strains grown in SC + 2% glucose. Dotted, dashed, and solid blue lines represent 3 different WHI5/whi5 clones. (F) Predicted effects of WHI5 dosage on cell size in a mathematical model of Start. Box and whisker plots show distribution of critical cell sizes predicted by the Start model published in [22] for simulated average Whi5 concentrations of 120 nM (corresponding to WHI5-GFP cells, left boxplot) and 85 nM (corresponding to WHI5-NRS1-GFP cells, right boxplot). All numerical values underlying panels C–F may be found in S7 Data. NRS1, Nitrogen-Responsive Start regulator 1; WT, wild-type; YNB+Pro, YNB + 0.4% proline + 2% glucose. (PDF) [file pbio.3001548.s006.pdf]

**A**

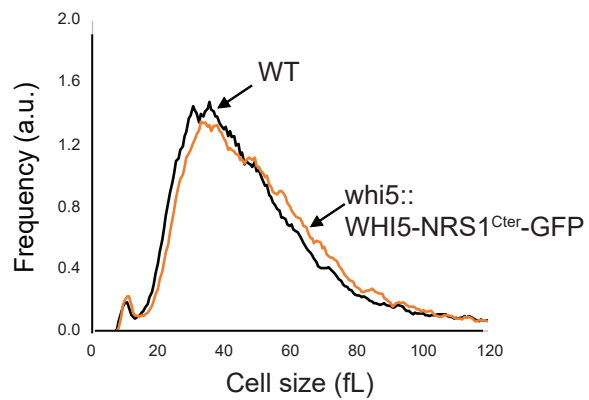

**B**

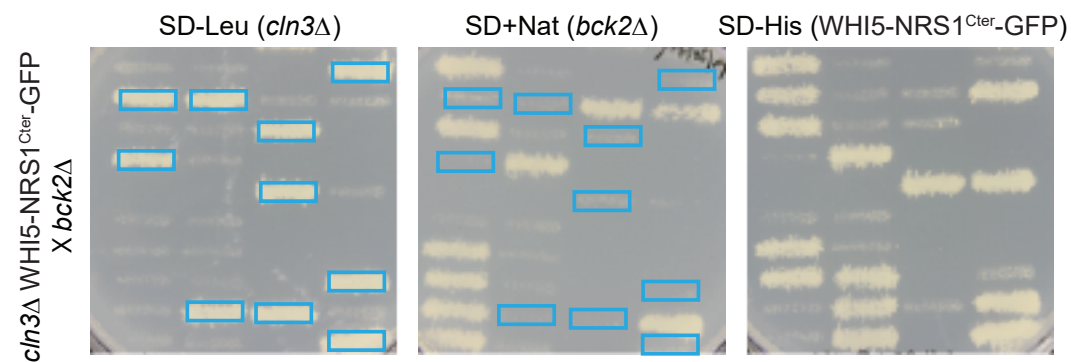

Supplement: S7 Fig — (A) Cell size distributions of WT and WHI5-NRS1Cter-GFP strains grown in SC+2% glucose determined on a Beckman Z2 Coulter counter. (B) Genotype of 10 tetrads from a cln3Δ whi5::WHI5-NRS1Cter-GFP X bck2Δ cross. For each tetrad, spore clone growth was assessed on SD-Leu (indicates cln3::LEU2), SC+NAT (indicates bck2::NATR), and SD-HIS (indicates whi5::WHI5-NRS1Cter-GFP-HIS3). Blue boxes indicate viable cln3Δ or bck2Δ spore clones. No viable cln3Δ bck2Δ double mutant clones were recovered. All numerical values underlying panel A may be found in S7 Data. NRS1, Nitrogen-Responsive Start regulator 1; WT, wild-type. (PDF) [file pbio.3001548.s007.pdf]
