# Supplementary material for: The microprotein Nrs1 rewires the G1/S transcriptional machinery during nitrogen limitation in budding yeast
Source: PLoS Biol. 2022 Mar 3;20(3):e3001548. doi: 10.1371/journal.pbio.3001548 (PMC8893695; doi:10.1371/journal.pbio.3001548)
Supplement: S2 Table — (DOCX) [file pbio.3001548.s009.docx]

**Table S2. Yeast strains and plasmids used in this study**

| **Strain name** | **Strain**  **number** | **Relevant**  **genotype** | **MAT** | **Source** |
| --- | --- | --- | --- | --- |
| Wild-type (WT) haploid | yMT1448 | *his3Δ1 leu2Δ0 met15Δ0 ura3Δ0* | **a** | (1) |
| WT diploid | yMT1450 | *his3/his3 leu2/leu2 ura3/ura3 MET15/met15 LYS2/lys2* | **a**/α | C. Roberts (gift) |
| *WHI5*/*whi5Δ *diploid, clone 1 | yMT5036 | *WHI5/whi5::kanMX* | **a**/α | This work |
| *WHI5*/*whi5Δ *diploid, clone 2 | yMT5037 | *WHI5/whi5::kanMX* | **a**/α | This work |
| *WHI5*/*whi5Δ *diploid, clone 3 | yMT5038 | *WHI5/whi5::kanMX* | **a**/α | This work |
| *cln3Δ bck2Δ GAL1-WHI5* | yMT4317 | *cln3::LEU2 bck2::natMX whi5::kanMX-pGAL1-WHI5 mfa1::MFA1pr-spHIS5+ can1Δ* |  | This work |
| Nrs1-GFP | yMT5039 | *nrs1:: NRS1-mGFPmut3-HIS3MX* | **a** | This work |
| Nrs1^13MYC^ | yMT4321 | *nrs1:: NRS1-13MYC-kanMX* | **a** | This work |
| Nrs1-WT GFP | yMT5040 | *nrs1::NRS1-GFP-natMX* | **-** | This work |
| *nrs1Δ* | BY2664 | *nrs1::kanMX* | **a** | (2) |
| *GAL1-NRS1* | yMT4323 | *lys2 nrs1::kanMX-pGAL1-NRS1* | **a** | This work |
| *cln3Δ* | yMT2125 | *cln3Δ::LEU2* | **a** | (3) |
| *bck2Δ* | BY6163 | *bck2Δ::kanMX* | **a** | (2) |
| *swi6Δ* | BY4131 | *swi6Δ::kanMX* | **a** | (2) |
| *swi4Δ* | BY6109 | *swi4Δ:: kanMX* | **a** | (2) |
| *swi4-ts* | yMT5044 | *trp1Δ63 ura3-52 lys2-801a ade2-107o his3Δ200 leu2Δ1 swi4-ts* |  | B. Andrews (gift) |
| *swi4-ts GAL1-NRS1* | yMT5047 | *trp1Δ63 ura3-52 lys2-801a ade2-107o his3Δ200 leu2Δ1 swi4-ts nrs1::kanMX-pGAL1-NRS1* |  | This work |
| *whi5Δ* | yMT3249 | *whi5Δ::kanMX* | **a** | (4) |
| *whi5Δ nrs1Δ* | yMT5042 | *whi5Δ::kanMX nrs1::natMX* | − | This work |
| Whi5-GFP | yMT5009 | *whi5::WHI5-mGFPmut3-HIS3MX* | **a** | (6) |
| *GAL1-NRS1 Whi5-GFP* | yMT5043 | *whi5::WHI5-mGFPmut3-HIS3MX kanMX-pGAL1-NRS1* | − | This work |
| Swi6-GFP | yMT5006 | *swi6::SWI6-mGFPmut3-HIS3MX* | **a** | (6) |
| Whi5^13MYC^ | yMT3248 | *whi5:: WHI5-13MYC-kanMX* | **a** | (4) |
| Whi5^HA^ | yMT4325 | *whi5:: WHI5-3HA-kanMX* | **a** | This work |
| *GAL1-NRS1* Whi5^HA^ | yMT4320 | *nrs1::kanMX-pGAL1-NRS1 whi5:: WHI5-3HA-HIS3MX* | − | This work |
| *HIS3* reporter strain | AH109 | *trp10901 leu2-3, 112 ura3-52 his3-200 gal4Δ gal80Δ lys2::GAL1_UAS_-GAL1_TATA_-HIS3 GAL2_UAS_-GAL2_TATA_-ADE2 ura3::MEL1_UAS_-MEL1_TATA_-lacZ* | **a** | Clontech (7) |
| WT (Fig 5, S5) | yMT5052 | *trp1Δ63 GAL2+ ura3-52 lys2-801a ade2-107o his3Δ200 leu2Δ1* |  | B. Andrews (gift) |
| *mbp1Δ* | yMT5046 | *trp1Δ63 GAL2+ ura3-52 lys2-801a ade2-107o his3Δ200 leu2Δ1 mbp1::TRP1* |  | B. Andrews (gift) |
| *swi4-ts mbp1Δ* | yMT5045 | *trp1Δ63 GAL2+ ura3-52 lys2-801a ade2-107o his3Δ200 leu2Δ1 swi4-ts mbp1::TRP1* |  | B. Andrews (gift) |
| *GAL1-NRS1 swi4-ts mbp1Δ clone C2* | yMT5048 | *trp1Δ63 GAL2+ ura3-52 lys2-801a ade2-107o his3Δ200 leu2Δ1 swi4-ts mbp1::TRP1 nrs1::kanMX-pGAL1-NRS1* |  | This work |
| *GAL1-NRS1 swi4-ts mbp1Δ clone C7* | yMT5049 | *trp1Δ63 GAL2+ ura3-52 lys2-801a ade2-107o his3Δ200 leu2Δ1 swi4-ts mbp1::TRP1 nrs1::kanMX-pGAL1-NRS1* |  | This work |
| *GAL1-NRS1 swi4-ts mbp1Δ clone C11* | yMT5050 | *trp1Δ63 GAL2+ ura3-52 lys2-801a ade2-107o his3Δ200 leu2Δ1 swi4-ts mbp1::TRP1 nrs1::kanMX-pGAL1-NRS1* |  | This work |
| *GAL1-NRS1 swi4-ts mbp1Δ clone C12* | yMT5051 | *trp1Δ63 GAL2+ ura3-52 lys2-801a ade2-107o his3Δ200 leu2Δ1 swi4-ts mbp1::TRP1 nrs1::kanMX-pGAL1-NRS1* |  | This work |
| Whi5-Nrs1-GFP | yMT5053 | *whi5::WHI5-NRS1-mGFPmut3-HIS3MX* | **a** | This work |
| *bck2Δ* | yMT2299 | *bck2Δ::natMX* |  | This work |
| *cln3Δ WHI5-NRS1-GFP* | yMT5055 | *cln3Δ::LEU2 whi5::WHI5-NRS1-mGFPmut3-HIS3MX* | **a** | This work |
| *cln3Δ bck2Δ WHI5-NRS1-GFP clone 1* | yMT5057 | *cln3Δ::LEU2 bck2Δ::natMX whi5::WHI5-NRS1-mGFPmut3-HIS3MX* | **-** | This work |
| *cln3Δ bck2Δ WHI5-NRS1-GFP clone 2* | yMT5058 | *cln3Δ::LEU2 bck2Δ::natMX whi5::WHI5-NRS1-mGFPmut3-HIS3MX* | **-** | This work |
| *cln3Δ bck2Δ WHI5-NRS1-GFP clone 3* | yMT5059 | *cln3Δ::LEU2 bck2Δ::natMX whi5::WHI5-NRS1-mGFPmut3-HIS3MX* | **-** | This work |
| *cln3Δ bck2Δ WHI5-NRS1-GFP clone 4* | yMT5060 | *cln3Δ::LEU2 bck2Δ::natMX whi5::WHI5-NRS1-mGFPmut3-HIS3MX* | **-** | This work |
| *cln3Δ bck2Δ WHI5-NRS1-GFP clone 5* | yMT5061 | *cln3Δ::LEU2 bck2Δ::natMX whi5::WHI5-NRS1-mGFPmut3-HIS3MX* | **-** | This work |
| WT *S. boulardii* | yMT5073 | MYA-796 | **-** | ATCC |
| *nrs1Δ S. boulardii* | yMT5074 | MYA-796 *nrs1Δ* | **-** | This work |
| *cln∆ strain* | yMT3743 | *cln1∆ ::hisG cln2∆  cln3∆ ::LEU2 MET-CLN2 ::TRP1* | **a** | (5) |
| *cln∆ strain GAL1-NRS1* | yMT5065 | *cln1∆ ::hisG cln2∆  cln3∆ ::LEU2 MET-CLN2 ::TRP1 pGAL1-NRS1-URA3* | **a** | This work |
| *mbp1∆* | yMT5098 | *mbp1∆::nat1* |  | This work |
| *mbp1∆ nrs1Δ* | yMT5099 | *mbp1∆::nat1 mbp1∆::kanMX* | **-** |  |
| Whi5-Nrs1^Cter^-GFP  Swi4^3FLAG^  Swi6^3FLAG^  Whi5^13MYC^ Swi4^3FLAG^  Whi5^13MYC^ Swi6^3FLAG^  Nrs1^13MYC^ Swi4^3FLAG^  Nrs1^13MYC^ Swi6-^3FLAG^ | yMT5100  yMT5123  yMT5124  yMT5125  yMT5126  yMT5127  yMT5128 | *whi5::WHI5-* NRS1^Cter^*-mGFPmut3-HIS3MX*  Nrs1^Cter^ *=* *KKHNPFYVPSEVVREMVKKHALNGRI*  *swi4::SWI4-3xFLAG-HIS3*  *Swi6::SWI6-3xFLAG-HIS3*  *whi5:: WHI5-13MYC-kanMX swi4::SWI4-3xFLAG-HIS3*  *whi5:: WHI5-13MYC-kanMX swi6::SWI6-3xFLAG-HIS3*  *nrs1:: NRS1-13MYC-kanMX swi4::SWI4-3xFLAG-HIS3*  *nrs1:: NRS1-13MYC-kanMX swi6::SWI6-3xFLAG-HIS3* | **a**  **a**  **a**  **a**  **a**  **a**  **a** | This work  This work  This work  This work  This work  This work  This work |
| **Plasmid name** | **Plasmid**  **number** | **Description** |  | **Source** |
| pVenus | pMT4642 | yEpVenus_URA: URA3 2μm plasmid, TDH3-promoter-driven expression of Venus (YFP) |  | (8) |
| pmCherry | pMT4643 | yEpGAP-Cherry: URA3 2μm plasmid, TDH3-promoter-driven expression of yEmRFP |  | (9) |
| pGAL1-NRS1 | pMT4644 | pGAL1-NRS1-HA URA3 2μ plasmid built in the pBG1805 backbone from Gelperin *et al.* 2005 |  | This work |
| pGAL1-WHI5 | pMT3445 | pGAL1-WHI5 HIS3 CEN |  | (4) |
| pGAL4DBD | pMT4645 | pGBKT7 cloning vector TRP1 2μm |  | Clontech |
| pGAL4DBD-UBE2G2 | pMT4646 | pGBKT7-UBE2G2 |  | This work |
| pGAL4DBD-NRS1 | pMT4647 | pGBKT7-NRS1 |  | This work |
| pGAL4DBD-Cter(NRS1) | pMT4648 | pGBKT7*-NRS1^83-99^* |  | This work |
| pGAL1-Whi5^12A^ | pMT3455 | pGAL1-WHI5^12A^ HIS3 CEN |  | (4) |
| pGAL1-SWI6^S4A^ | pBD1756 | SWI6^S4A^ LEU2 2μm plasmid, derived from pBD176 |  | (10) |
| pGZ110-sgRNA_NRS1 | pMT4703 | pGZ110-Cas9-sgRNA_NRS1-amdSYM |  | This work |
| GST-YLR053c | pMT4575 | pGEX-2T-GST-NRS1. Bacterial expression |  | This work |

* All strains are congenic with yMT1448 (S288C background) unless otherwise indicated

**References**

1. Winzeler E.A. et al. (1999) Functional characterization of the S. cerevisiae genome by gene deletion and parallel analysis. Science 285(5429):901-6. doi: 10.1126/science.285.5429.901.

2. Giaever G. et al. (2002) Functional profiling of the Saccharomyces cerevisiae genome. Nature 418(6896):387-91. doi: 10.1038/nature00935.

3. Jorgensen P. et al. (2002) Systematic identification of pathways that couple cell growth and division in yeast. Science 297(5580):395-400. doi: 10.1126/science.1070850.

4. Costanzo M. et al. (2994) CDK activity antagonizes Whi5, an inhibitor of G1/S transcription in yeast. Cell 117(7):899-913. doi: 10.1016/j.cell.2004.05.024.

5. Irniger S. and Nasmyth K. (1997) The anaphase-promoting complex is required in G1 arrested yeast cells to inhibit B-type cyclin accumulation and to prevent uncontrolled entry into S-phase. J Cell Sci. 110 ( Pt 13):1523-31.

6. Dorsey S. et al. (2018) G1/S Transcription Factor Copy Number Is a Growth-Dependent Determinant of Cell Cycle Commitment in Yeast. Cell Syst. 6(5):539-554.e11. doi: 10.1016/j.cels.2018.04.012.

7. James P. et al. (1996) Genomic libraries and a host strain designed for highly efficient two-hybrid selection in yeast. Genetics 144(4):1425-36. doi: 10.1093/genetics/144.4.1425.

8. Bilsland E. et al. (2013) Yeast-based automated high-throughput screens to identify anti-parasitic lead compounds. Open Biol. 2013 3(2):120158. doi: 10.1098/rsob.120158.

9. Keppler-Ross S. et al. (2008) A new purple fluorescent color marker for genetic studies in Saccharomyces cerevisiae and Candida albicans. Genetics 179(1):705-10. doi: 10.1534/genetics.108.087080.

10. Sidorova J.M. et al. (1995) Cell cycle-regulated phosphorylation of Swi6 controls its nuclear localization. Mol Biol Cell. 6(12):1641-58. doi: 10.1091/mbc.6.12.1641.
