# Supplementary material for: The microprotein Nrs1 rewires the G1/S transcriptional machinery during nitrogen limitation in budding yeast
Source: PLoS Biol. 2022 Mar 3;20(3):e3001548. doi: 10.1371/journal.pbio.3001548 (PMC8893695; doi:10.1371/journal.pbio.3001548)

Figure 2B, original WB

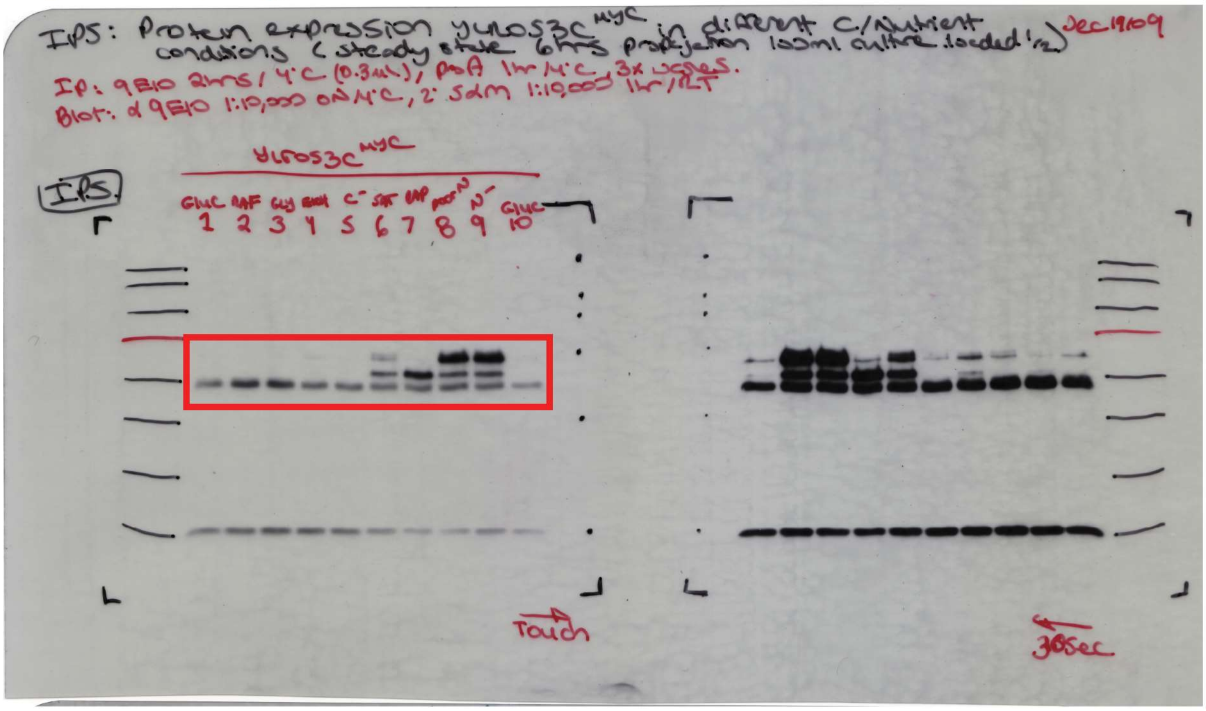

Figure 2C top, original WB

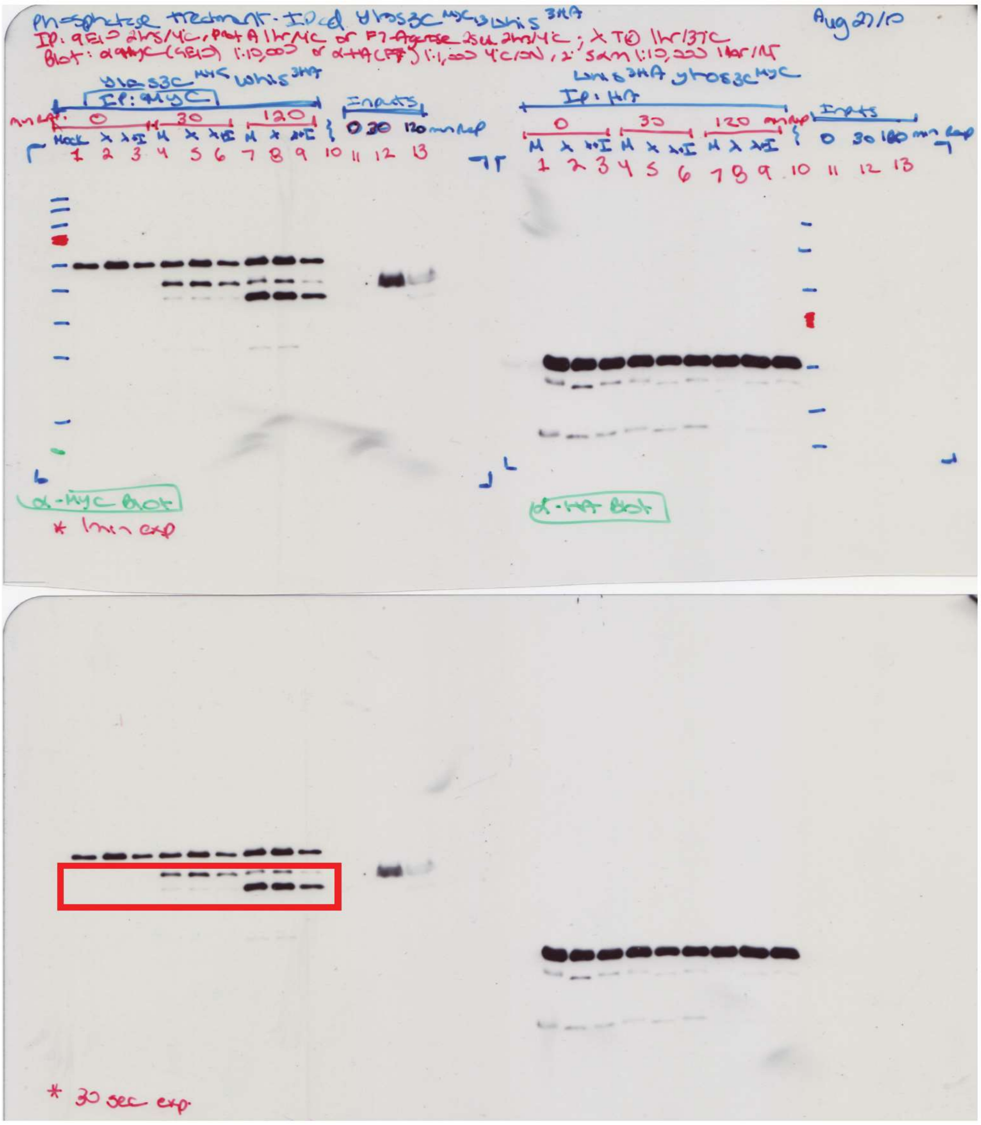

Figure 2C bottom, original WB

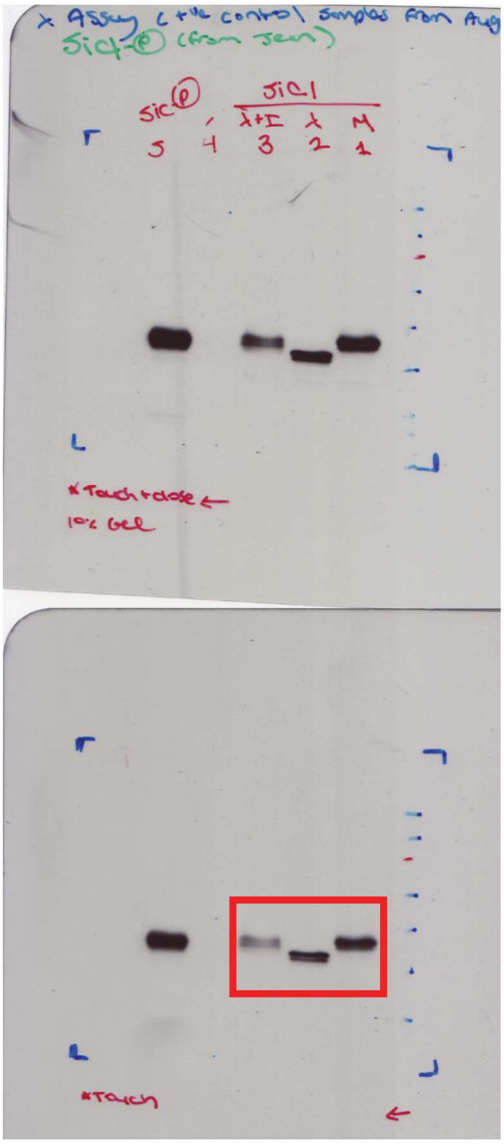

Figure 5A, original WBs

Panel 1: Anti-MYC

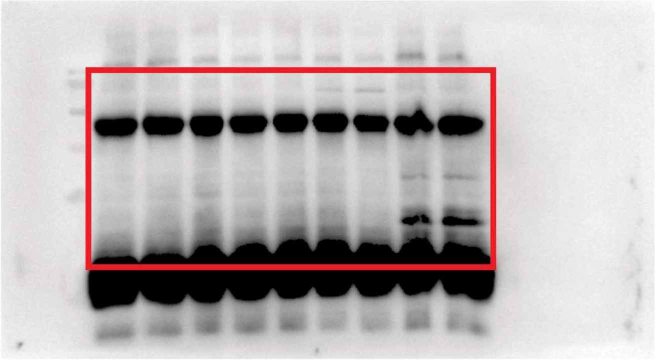

Panel 2: Anti-FLAG

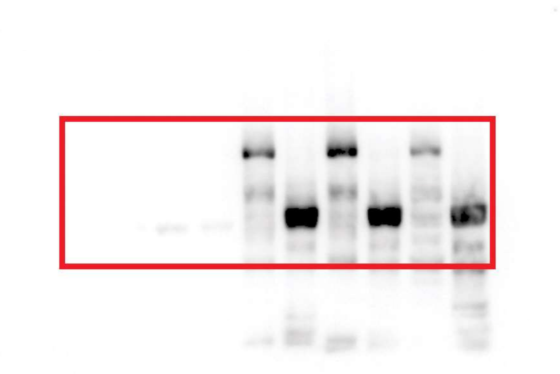

Panel 3: Anti-MYC

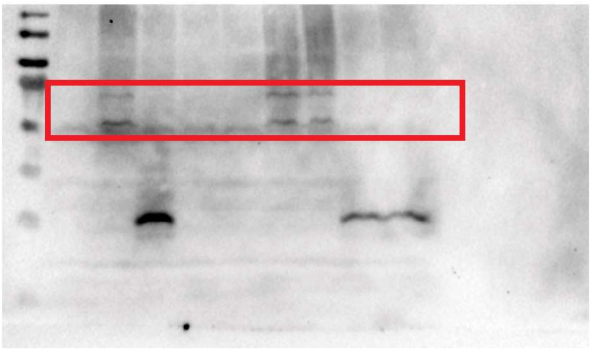

Panel 4: Anti-MYC

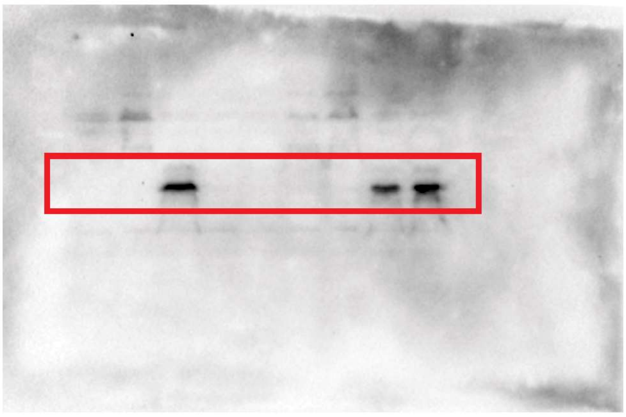

Panel 5: Anti-FLAG

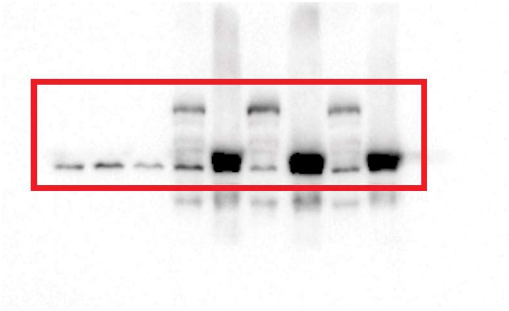

Panel 6: Anti-Pgk1

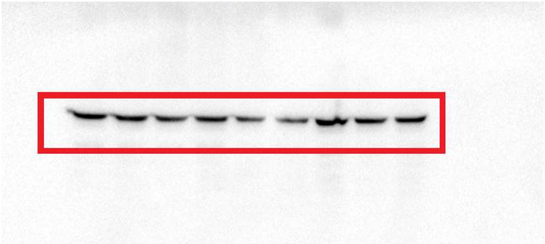

Figure 5B top left, original blot

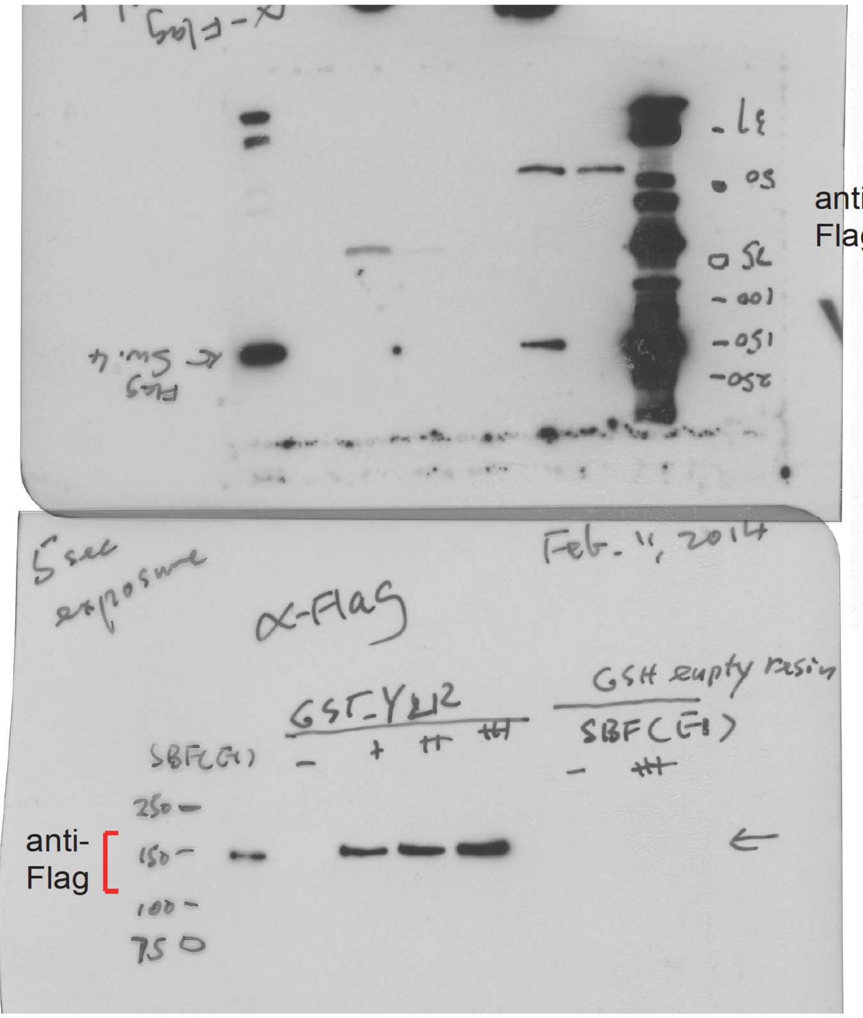

Figure 5B top right, original blot

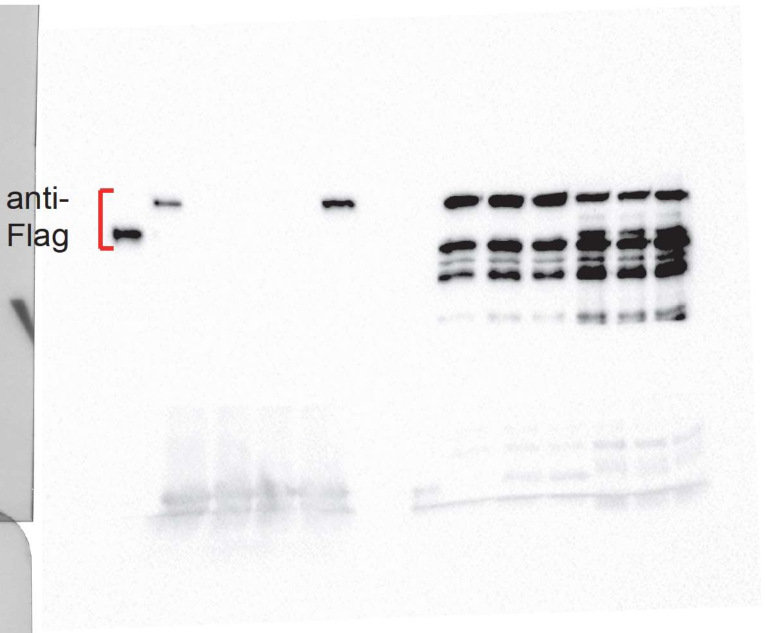

Figure 5B bottom left, original blot

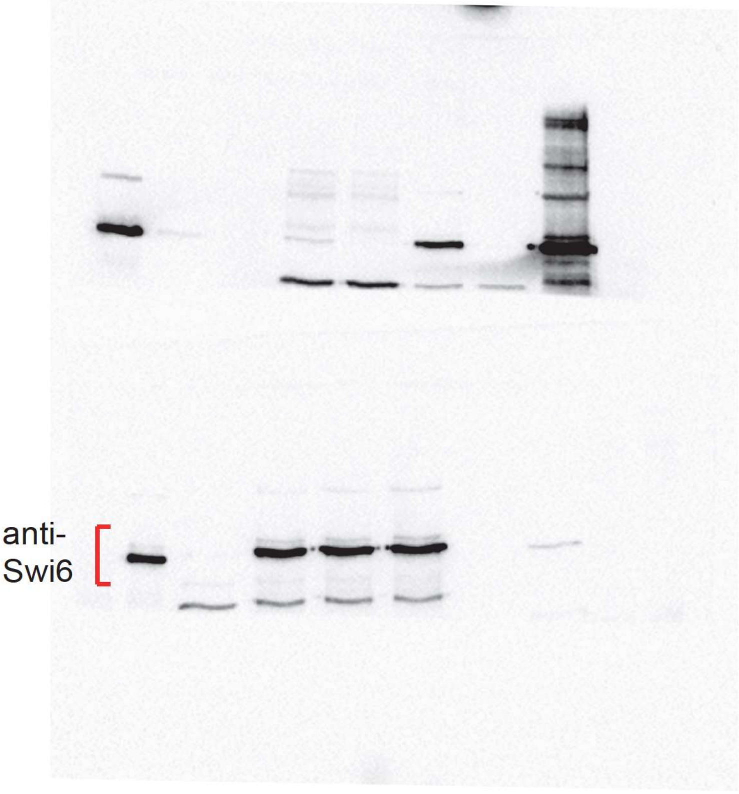

Figure 5B bottom right, original blot

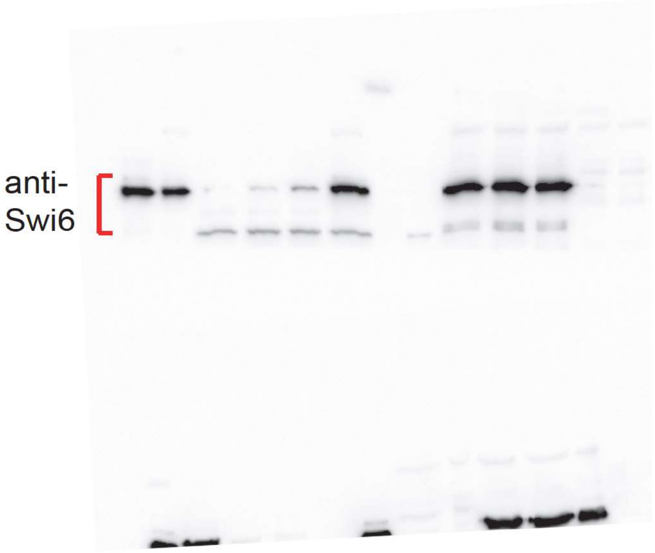

Figure 5C top, original WB

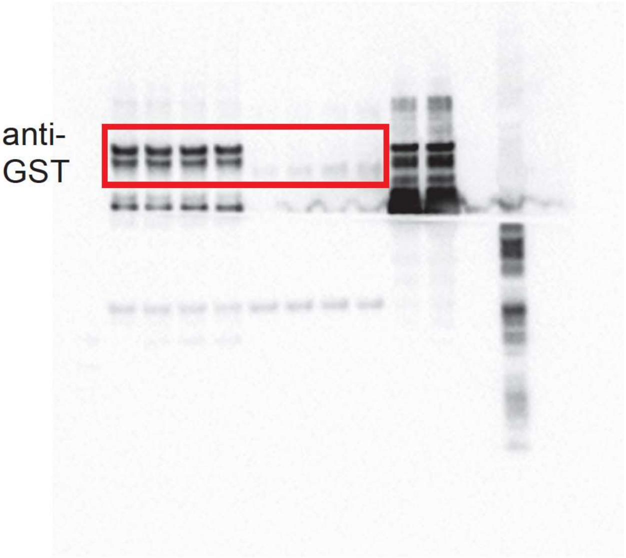

Figure 5C middle, original blot

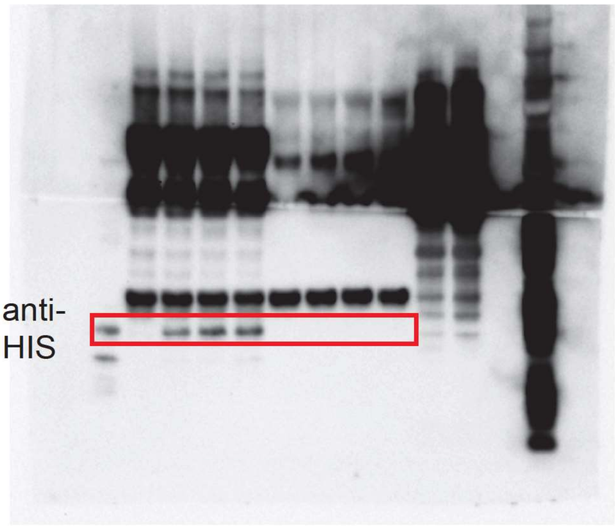

Figure 5C bottom, original Ponceau

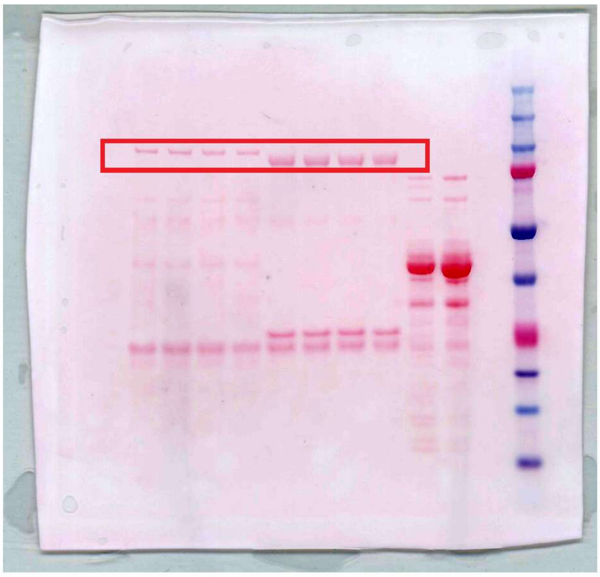

Figure S1C, original WB

anti-Myc

Input

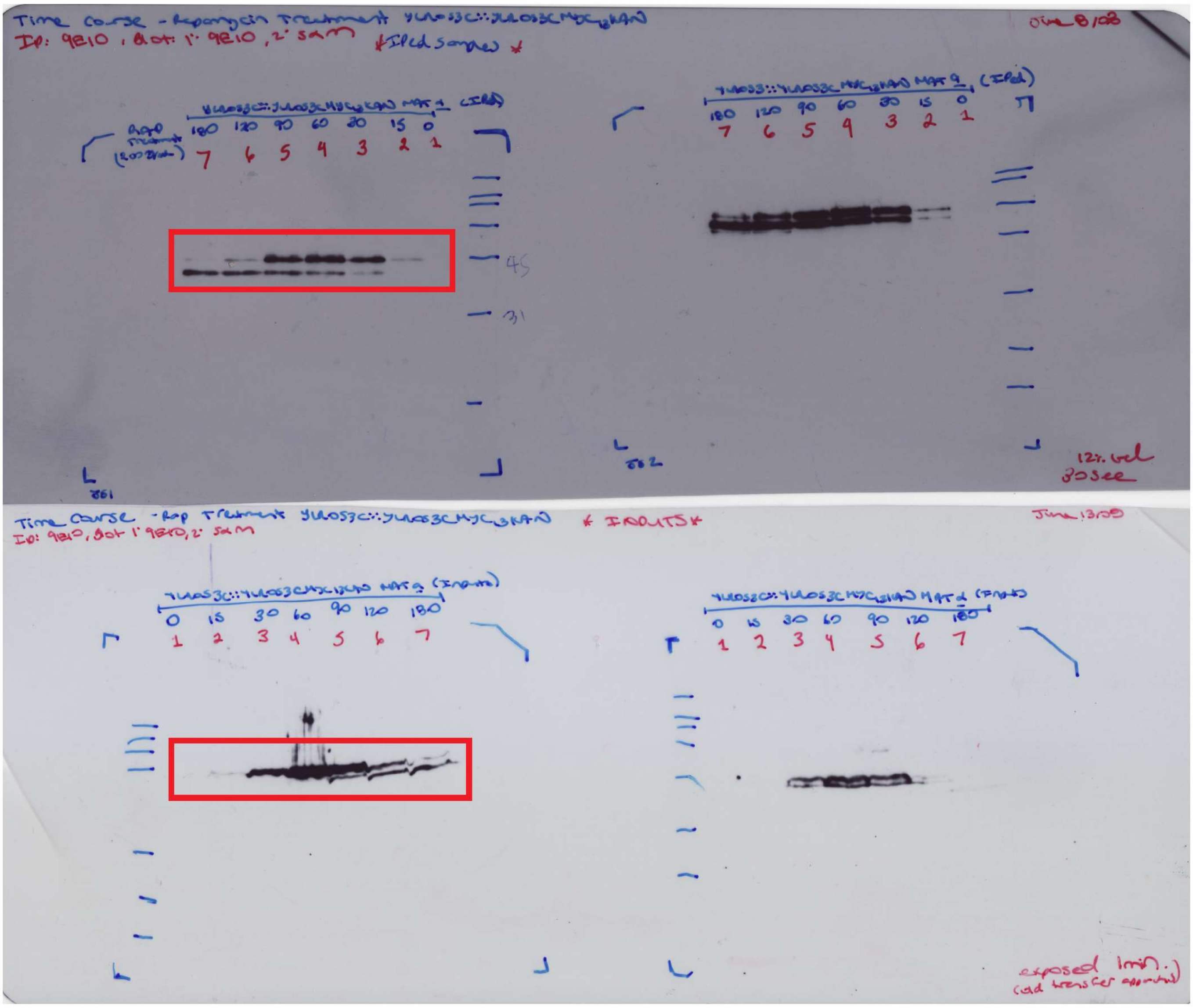

Figure S1F, original WB

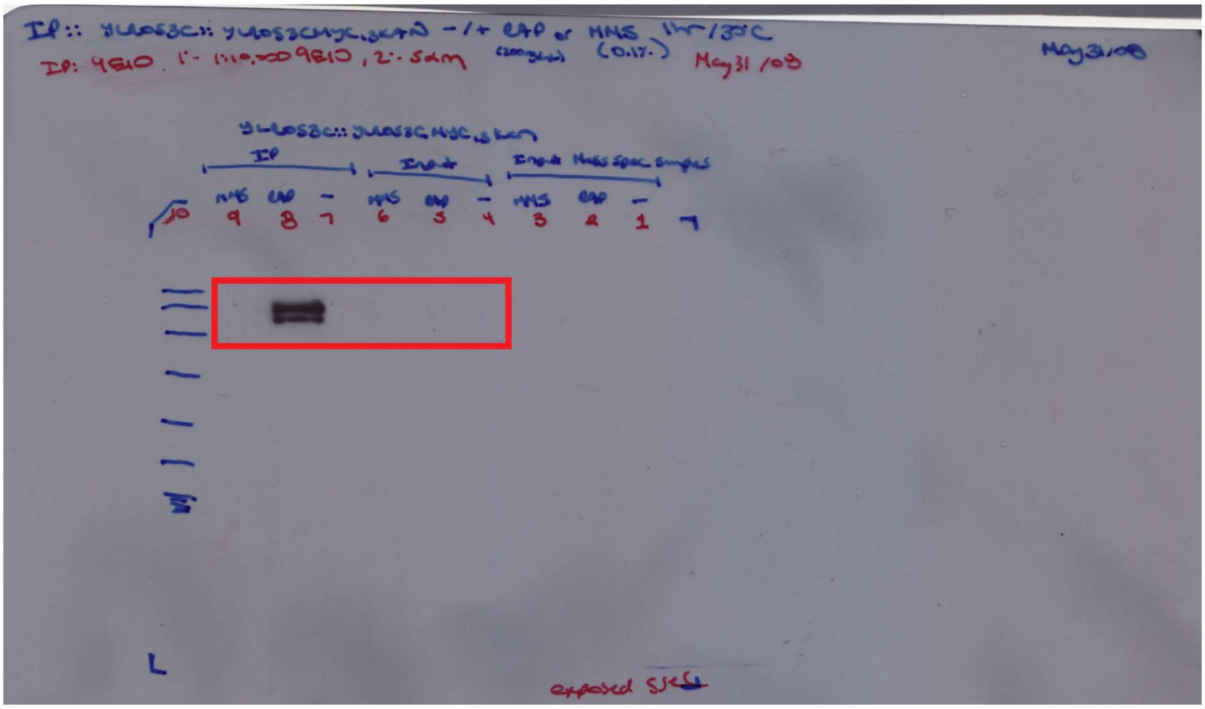

Figure S3B, original WBs

Panel 1: Anti-MYC

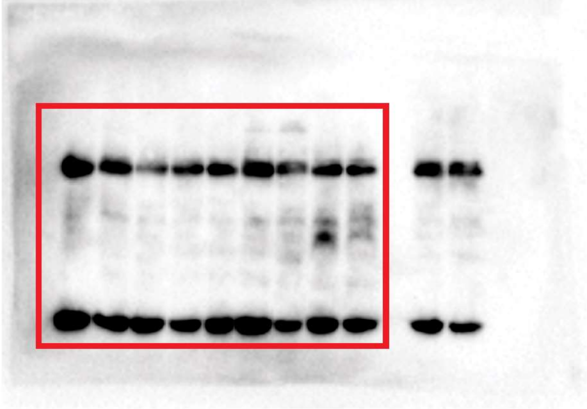

Panel 2: Anti-FLAG

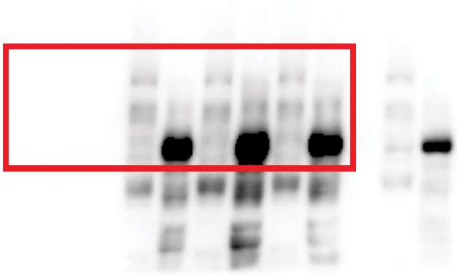

Panel 3: Anti-MYC

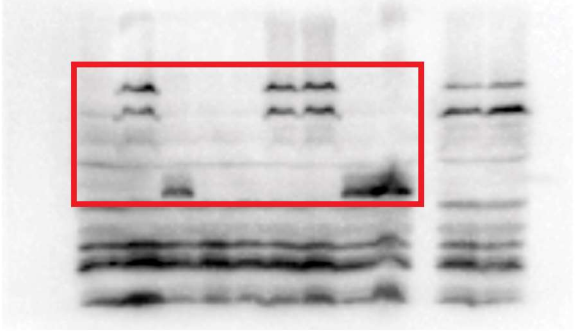

Panel 4: Anti-FLAG

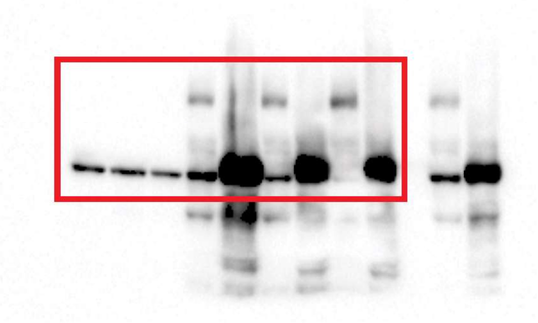

Panel 5: Anti-Pgk1

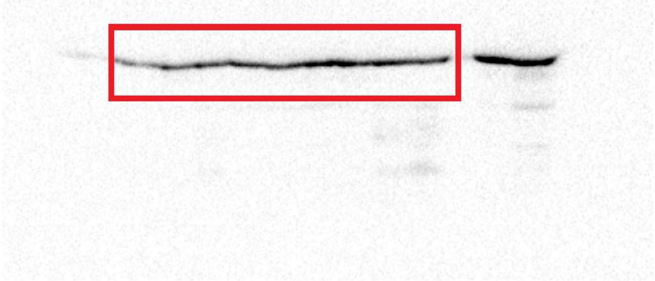

Figure S3C, original WBs

Panel 1: Anti-Swi4

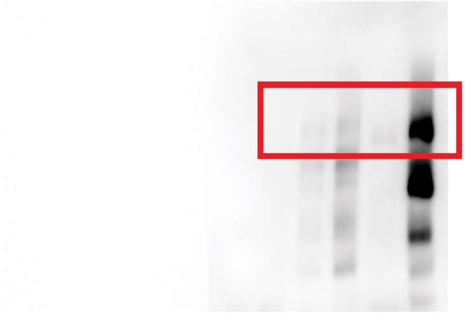

Panel 2: Anti-Swi6

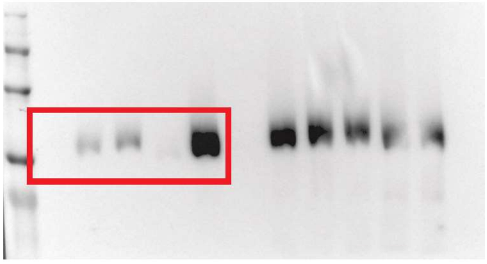

Panel 3: Anti-MYC

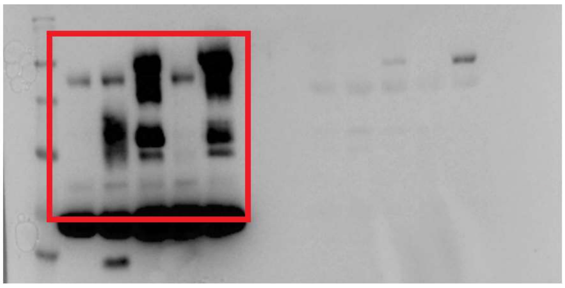

Panel 4: Anti-Swi4

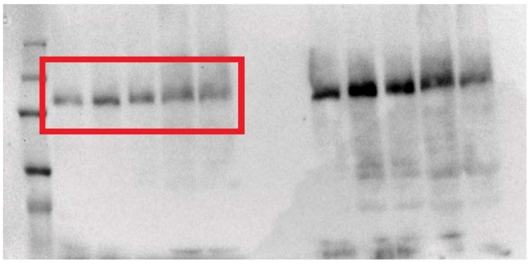

Panel 5: Anti-Swi6

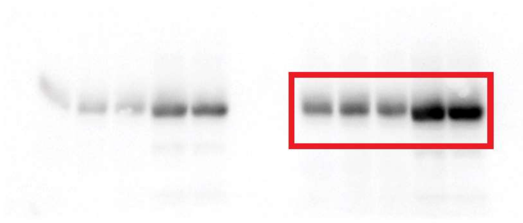

Panel 6: Anti-MYC

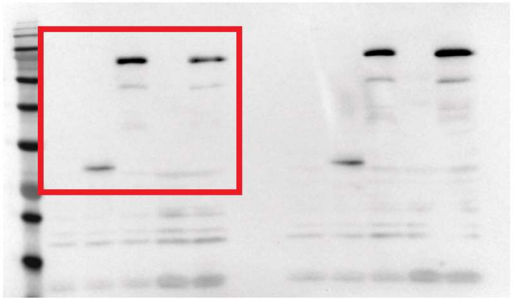

Figure S4C - orignal blots, Whi5 and Swi6 detection

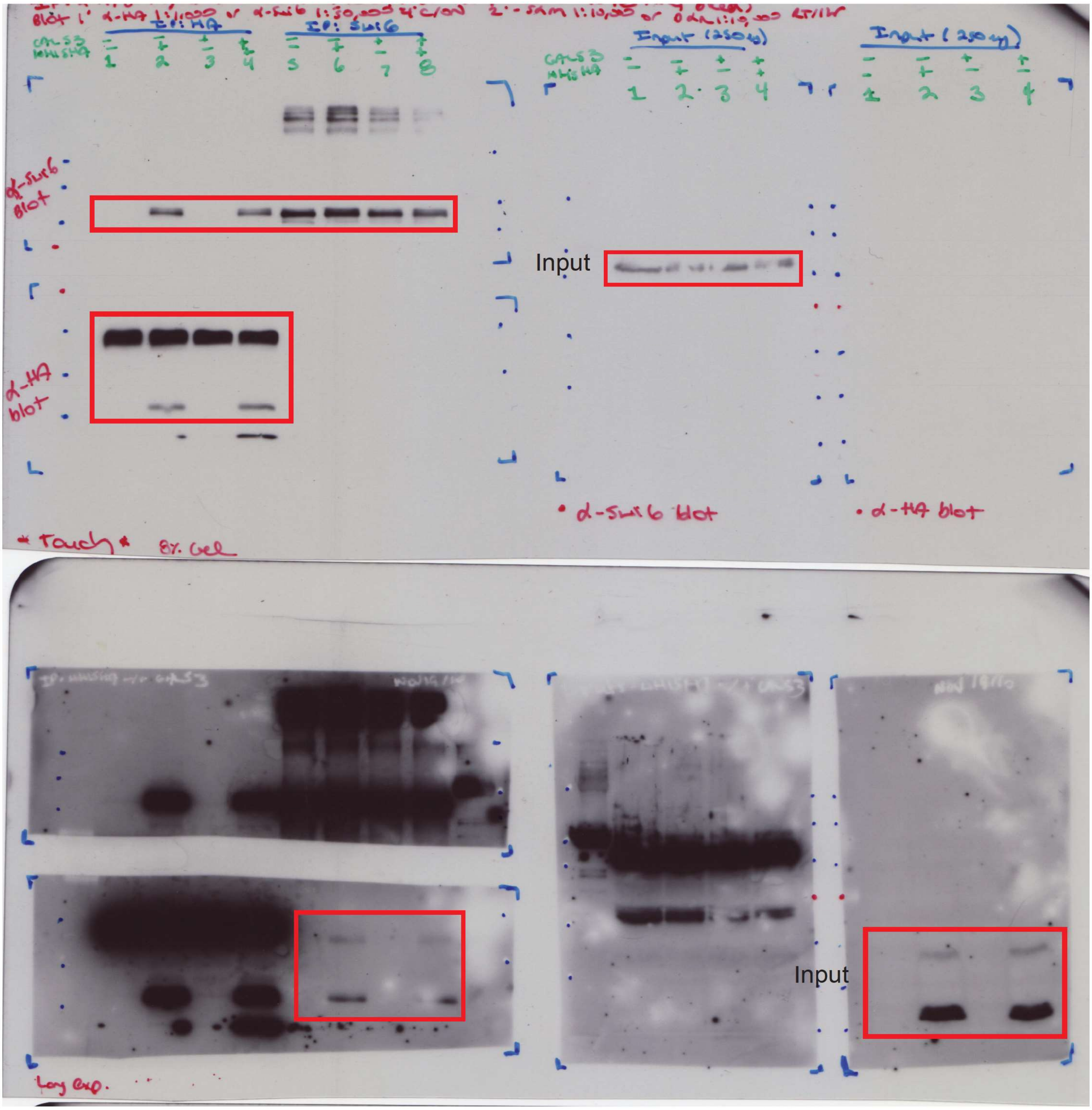

Figure S4C - original WB, anti-Swi4

IP-
HA
and
IP-
Swi6

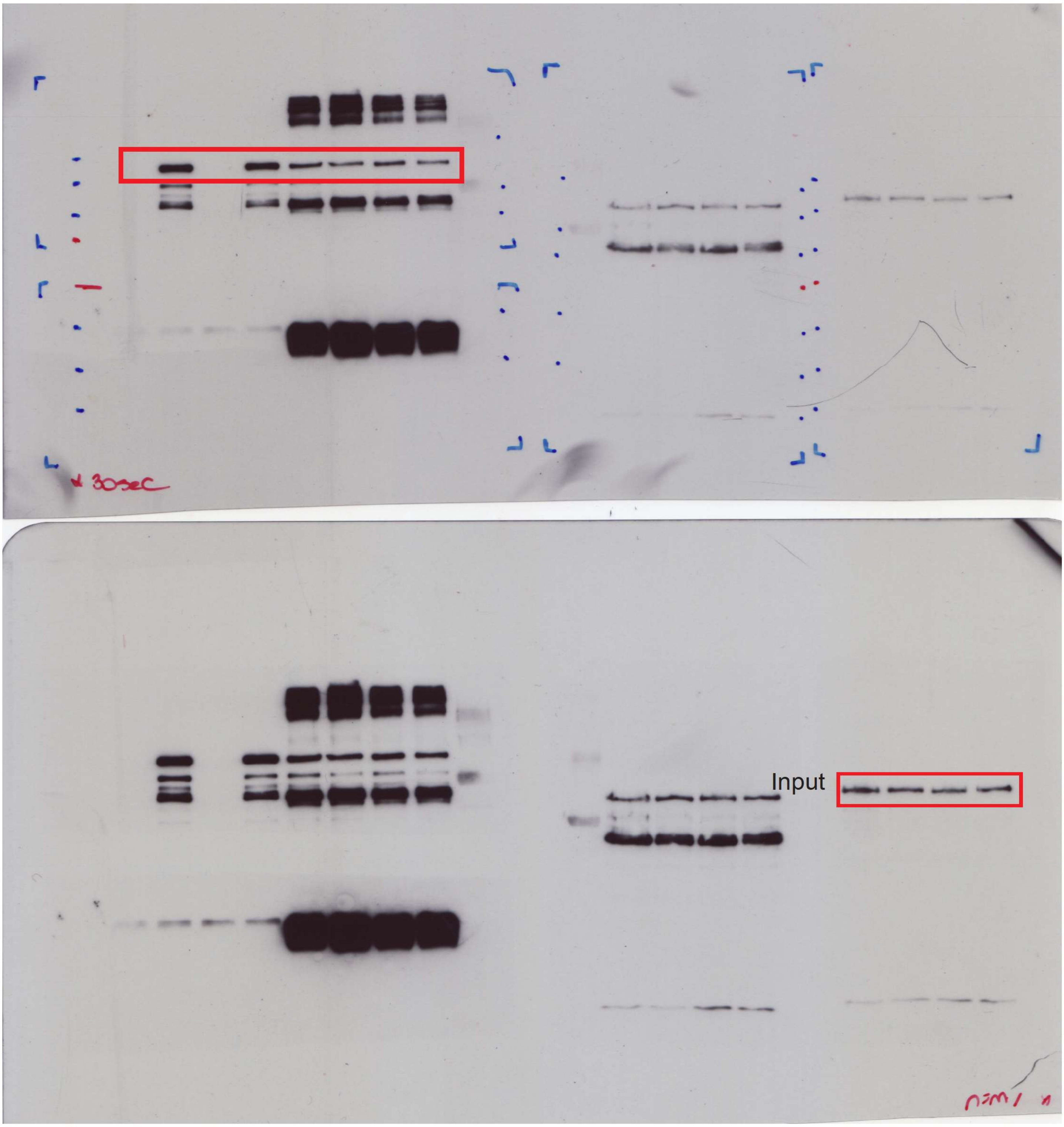

Figure S4D top, original WB, anti-HA

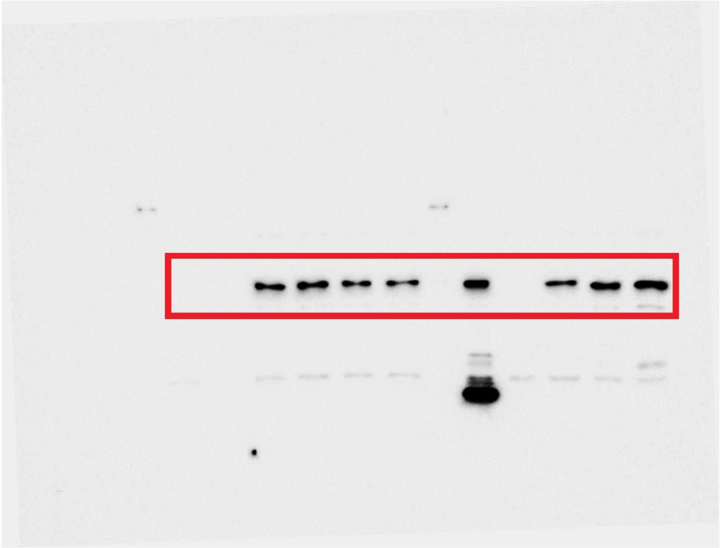

Figure S4D middle, original WBanti-GST

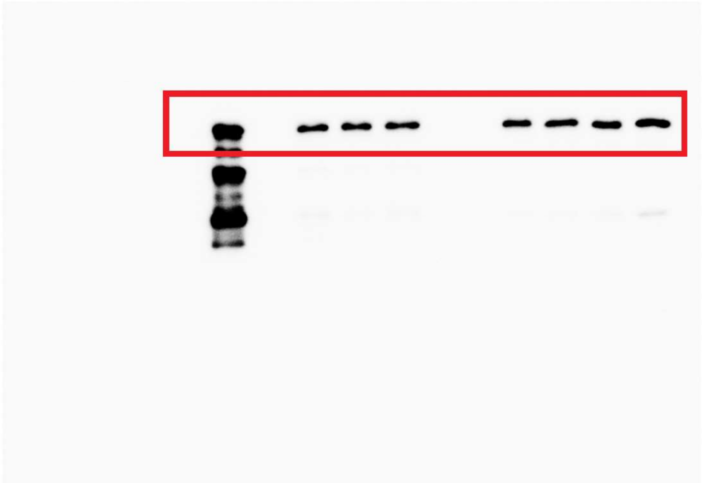

Figure S4D bottom, original Coomassie gel

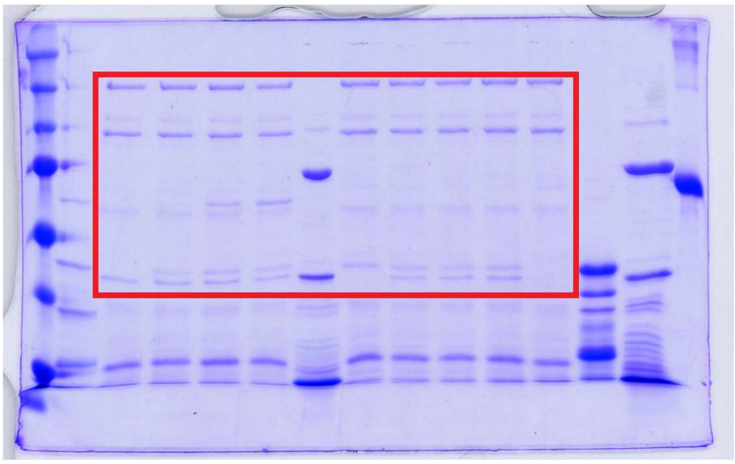

Figure S6A, original WB, anti-GFP

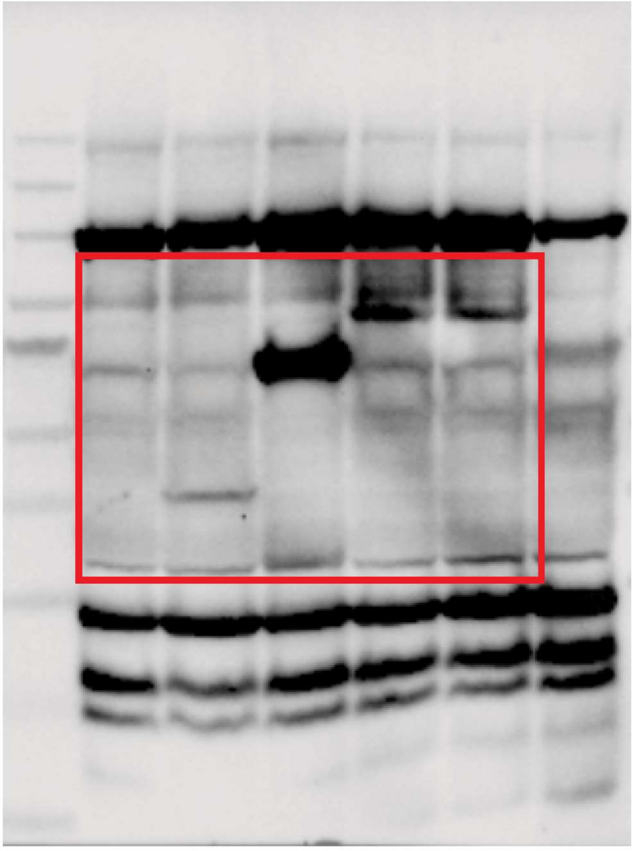

Supplement: S1 Raw Images — (PDF) [file pbio.3001548.s014.pdf]
