# Supplementary material for: The microprotein Nrs1 rewires the G1/S transcriptional machinery during nitrogen limitation in budding yeast
Source: PLoS Biol. 2022 Mar 3;20(3):e3001548. doi: 10.1371/journal.pbio.3001548 (PMC8893695; doi:10.1371/journal.pbio.3001548)
Supplement: S1 Text — (PDF) [file pbio.3001548.s022.pdf]

Supplemental Information

for

The microprotein Nrs1 rewires the G1/S transcriptional machinery during nitrogen limitation in budding yeast

Sylvain Tollis[1,2,*, †], Jaspal Singh[3,†] , Roger Palou[2,#], Yogitha Thattikota[2,‡,#], , Ghada Ghazal[2], Jasmin Coulombe-Huntington[2], Xiaojing Tang[3], Susan Moore[2], Deborah Blake[2], Eric Bonneil[2], Catherine A. Royer[4], Pierre Thibault[2] and Mike Tyers[2,*]

[1] Institute of Biomedicine, University of Eastern Finland, Kuopio, Finland

[2] Institute for Research in Immunology and Cancer, University of Montréal, Montréal, Quebec, Canada

[3] Lunenfeld-Tanenbaum Research Institute, Mount Sinai Hospital, Toronto, Ontario, Canada

[4] Department of Biological Sciences, Rensselaer Polytechnic Institute, Troy, NY, USA

[†] equal contribution to this work as first and second authors

[#] equal contribution to this work as third and fourth authors

[‡] Present address: Montreal Neurological Institute, McGill University, Montréal, Quebec, Canada; Department of Neurology and Neurosurgery, McGill University, Montreal, Quebec, Canada

* To whom correspondence should be addressed: sylvain.tollis@uef.fi ; md.tyers@umontreal.ca

Supplemental Methods

Immunoprecipitation and mass spectrometry analysis

Untagged and *NRS1*^{13MYC} cell pellets from 100 mL of culture at OD_{600} = 1 were lysed in lysis buffer supplemented with protease inhibitors using a freezer mill. Half of the final lysate (0.5 mL) was incubated for 1h with 50 µL of GammaBind plus Sepharose beads (GE Healthcare) and 5 µL of anti-MYC antibody (Gentex) to capture protein complexes. The other half was incubated with beads only and served as a negative control. Samples were washed four times and beads were resuspended in 50 µL of 2X lysis buffer. Samples were separated on BioRad precast gels and the entire gel line for each sample was prepared for mass spectrometry at the CAPCA core facility (IRIC, https://capca.iric.ca/). Gels were destained in 50% MeOH (Sigma-Aldrich), shrunk in 50% acetonitrile (ACN), reconstituted in 50 mM ammonium bicarbonate with 10 mM TCEP [Tris(2-carboxyethyl)phosphine hydrochloride; Thermo Fisher Scientific] and vortexed for 1 h at 37°C. Chloroacetamide (Sigma-Aldrich) was added for alkylation to a final concentration of 55 mM. Samples were vortexed for another hour at 37°C. Trypsin (1 µg) was added and digestion performed for 8 h at 37°C. Peptides were extracted with 90% ACN, dried down, solubilized in 5% ACN-0.2% formic acid (FA) and loaded on a C4 guard column (Optimize Technologies) connected directly to the switching valve. Separation was on a home-made reversed-phase column (150-µm i.d. by 180 mm) with a 56 min gradient from 10 to 30% ACN-0.2% FA at 600-nL/min flow rate on an Easy-nLC 1200 connected to an Q-Exactive HF Biopharma (Thermo Fisher Scientific, San Jose, CA). Each full MS spectrum acquired at a resolution of 120,000 was followed by 15 tandem-MS (MS-MS) spectra on the most abundant multiply charged precursor ions. Tandem-MS experiments were performed using collision-induced dissociation (HCD) at a collision energy of 27%. Data were processed using PEAKS X (Bioinformatics Solutions, Waterloo, ON) and the Uniprot yeast database. Mass tolerances on precursor and fragment ions were 10 ppm and 0.01 Da, respectively. Variable selected post-translational modifications were carbamidomethyl (C), oxidation (M), deamidation (NQ), acetyl (N-ter) and phosphorylation (STY). The data were visualized with Scaffold 4.8.9 (protein threshold, 99%, with at least 2 peptides identified and a false-discovery rate [FDR] of 1% for peptides). Hit proteins specific to Nrs1^{13MYC} immunoprecipitates were obtained by subtracting proteins identified in the control sample and then filtering the candidate hits against the CRAPome database [1].

Competitive growth assays

Wild type (BY4741) and *nrs1::KanMX6* strains were transformed with 2 μm plasmids expressing Venus and mCherry from the *TDH3* promoter and bearing a *URA3* selection marker [2]. Single colonies of Venus and mCherry strains were inoculated in SC-URA + 2% glucose for 24h growth to saturation. Cell densities in each saturated culture were measured using a Beckman Z2 Coulter counter and an equal number of green and red cells of the two different strains (about 200μL of saturated culture per strain) were diluted in 5 mL of water. In parallel, an equal number of each individual strain was diluted in 5 mL of water as a fluorescence intensity control. 50 μL of each diluted culture was inoculated in triplicate into 5 mL SC-URA + 2%glucose or YNB + Leu + His + Met +0.4% Pro + 2% glucose, as indicated. Cultures were transferred to a 30°C rotary incubator and grown for 72h. Venus and mCherry fluorescence was measured for each culture with a Tecan M1000 plate reader (excitation 515nm and 587nm, respectively; emission 528nm and 610nm, respectively; 5 nm bandpass filters). The gain of the instrument was optimized using the single colour cultures to ensure signal linearity within the range of measurement. As a first approximation, the measured fluorescence signals in the red and green channels yield, respectively: $F_{red} = N_{red} * \varepsilon_{Cherry}$, $F_{green} = N_{green} * \varepsilon_{Venus}$, where N_{red} and N_{green} are the total number of red and green cells within the culture, and ε_{Cherry} and ε_{Venus} the amount of fluorescence emitted per cell in each channel. These equations can be rewritten in terms of the total number of cells in the culture, N_0, and the fractions f_{red} and f_{green} of red and green cells:

$$F_{red} = N_0 * f_{red} * \varepsilon_{Cherry} = N_0 * (1 - f_{green}) * \varepsilon_{Cherry}$$

$$F_{green} = N_0 * (1 - f_{red}) * \varepsilon_{Venus} = N_0 * f_{green} * \varepsilon_{Venus}$$

Hence, given a measurement of the fluorescence signals and the total number of cells in a mix, it is sufficient to know ε_{Cherry} and ε_{Venus} in all strain backgrounds. Coefficients were calculated in each strain background separately, using the single colour control cultures, where f_{red} and f_{green} = 0 or 1, and for which the residual fluorescence in the other channel (e.g., red channel for a Venus-coloured strain) was negligible (~0.1-0.5% at most). In principle, it is possible that a fluorescent protein *per se* may cause a change in cell fitness. To rule out this possibility, we also cross-analyzed complementary mixes, e.g., strain 1 tagged with mCherry and strain 2 tagged with Venus vs strain 1 tagged with Venus and strain 2 tagged with mCherry. For such mixes, if we denote f_1 the fraction of strain 1, the independence of f_1 with respect to colouring yields $F_{red,1} = N_{0,1} * f_1 * \varepsilon_{Cherry}$ and $F_{red,2} = N_{0,2} * (1 - f_1) * \varepsilon_{Cherry}$ from which we get:

$$f_1 = \frac{F_{red,1}/N_{0,1}}{F_{red,1}/N_{0,1} + F_{red,2}/N_{0,2}}$$

or, equivalently

$$f_1 = \frac{F_{green,2}/N_{0,2}}{F_{green,1}/N_{0,1} + F_{green,2}/N_{0,2}}$$

The data presented on panel E in S2 Fig was derived using this analysis method, where each F/N ratio was averaged over 3 replicate cultures in each experiment. This method provided two measurements of the same fraction, based on analysis of the red and green fluorescence separately, and independently of renormalization to fluorescent signal in control cultures. With good accuracy (~2-3%), fractions derived using the green and red channels were identical. These values were also in good agreement with fractions derived using ε_{Cherry} and ε_{Venus} coefficients calculated in each strain background separately, using the single colour control cultures.

Supplemental References

1. Mellacheruvu D, Wright Z, Couzens AL, Lambert JP, St-Denis NA, Li T, et al. The CRAPome: a contaminant repository for affinity purification-mass spectrometry data. Nat Methods. 2013;10(8):730-6. Epub 2013/08/08. doi: 10.1038/nmeth.2557. PMID: 23921808; PMCID: PMC3773500.

2. Bilsland E, Sparkes A, Williams K, Moss HJ, de Clare M, Pir P, et al. Yeast-based automated high-throughput screens to identify anti-parasitic lead compounds. Open Biol. 2013;3(2):120158. Epub 2013/03/01. doi: 10.1098/rsob.120158. PMID: 23446112; PMCID: PMC3603448.

3. Dorsey S, Tollis S, Cheng J, Black L, Notley S, Tyers M, et al. G1/S Transcription Factor Copy Number Is a Growth-Dependent Determinant of Cell Cycle Commitment in Yeast. Cell Syst. 2018;6(5):539-54 e11. Epub 2018/05/25. doi: 10.1016/j.cels.2018.04.012. PMID: 29792825.
