# Supplementary material for: The microprotein Nrs1 rewires the G1/S transcriptional machinery during nitrogen limitation in budding yeast
Source: PLoS Biol. 2022 Mar 3;20(3):e3001548. doi: 10.1371/journal.pbio.3001548 (PMC8893695; doi:10.1371/journal.pbio.3001548)
Supplement: Attachment — Submitted filename: Response_to_Reviewers.pdf [file pbio.3001548.s024.pdf]

We are glad that all reviewers found that we have adequately addressed their concerns and support publication of our manuscript in PLOS Biology. A point by point response is provided below, in response to their final comments.

<u>Response to Reviewer #1:</u>

- "The authors have adequately addressed my concerns and provided new data regarding the growth defect of nrs1 in nitrogen-poor media. However, these important new findings should be in the paper, not in the supplement (figures S2F and S2G). Otherwise it is ready for publication."

Again, we would like to thank Reviewer #1 for her/his constructive remarks. We believe that following the reviewer's suggestions has made our manuscript stronger, and strengthened our initial conclusions.

In addition, we agree with Reviewer #1 that the growth defect due to *NRS1* deletion in nitrogen-poor media (originally Figs. S2F-G) is an important finding. We have therefore inserted this result in the revised version of the manuscript as a new main Figure 4.

<u>Response to Reviewer #2:</u>

- "The new experiments, in the prototrophic background and in the double mutant with anothe SBF component, support a physiological role. The authors did a good job on the revisions, and I support publication of the manuscript."

We are glad that Reviewer #2 has been convinced by our additional experiments and data, and would like to thank him/her for his/her constructive remarks. We believe that following the reviewer's suggestions has made our manuscript stronger, and strengthened our initial conclusions.

<u>Response to Reviewer #3, Sergio Moreno:</u>

- "The authors has answered satisfactorily most of my criticisms and have performed additional experiments to support their conclusions. Therefore, I am happy to accept this revised version of the manuscript for publication in PLOS BIOLOGY."

We are glad that Reviewer #3 Professor Sergio Moreno has been convinced by our additional experiments and data, and would like to thank him again for his constructive criticisms on our manuscript. Following Pr. Moreno and other reviewer's suggestions for additional experiments has allowed to strengthen our initial conclusions and improve our manuscript.
