# Supplementary material for: The microprotein Nrs1 rewires the G1/S transcriptional machinery during nitrogen limitation in budding yeast
Source: PLoS Biol. 2022 Mar 3;20(3):e3001548. doi: 10.1371/journal.pbio.3001548 (PMC8893695; doi:10.1371/journal.pbio.3001548)
Supplement: Attachment — Submitted filename: Response_to_Reviewers.docx [file pbio.3001548.s023.docx]

We would like to thank the reviewers for their positive feedback and for constructive remarks. We have followed the reviewers’ suggestions and performed new experiments and analyses that have strengthened our initial conclusions. A point by point response is provided below.

**Response to Reviewer #1:**

*- “These authors are interested in identifying over-expressed genes that enable cells to Start the yeast cell cycle in the absence of two positive regulators (Cln3 and Bck2) and the presence of an excess of the negative regulator (Whi5). They screened a library that ectopically expresses genes at a very high level for rescue of this strain, and found 12 candidates. Two were expected (Cln1 and Cln3) and the others were either unknown functions or have no direct connection to Start at the moment. Among these is NRS1, which is an uncharacterized gene with promising characteristics in that it directly associates with the G1/S transcription complex SBF (Swi4/Swi6) and can function as a transcription activator when tethered to DNA. Nrs1 is only detected in nitrogen limitation or TOR inhibition (rapamycin treatment), hence the name Nitrogen Responsive Start regulator. Chromatin IP shows only a two-fold increase in Nrs1 on SBF target promoters that is rapamycin-dependent, so maybe it binds these promoters, but there is no transcription data to support that it activates these genes. “*

The reviewer's concern that we did not directly demonstrate activation of G1/S gene transcription by NRS1 is well taken. To address this key issue, we performed RNA-seq measurements of genome-wide transcription profiles as a time-course following *GAL1-NRS1* induction. These experiments definitively demonstrated that *NRS1* expression induces the transcription of 126 of the 139 SBF/MBF-regulated genes previously defined by the Aldea and Futcher groups (Ferrezuelo et al., 2010). By ranking genes in descending order of fold induction, *CLN2* ranked 12^th^ and *PCL1* 85^th^, consistent with our original demonstration the Nrs1 can be detected on these two promoters by ChIP. The RNA-seq data has been added as a new Figure 5C, and presented in parallel with ChIP data and RICS data from the initial submission (formerly Figure 4C, 4D, now Figure 5A, 5B). A new section describing the RNA-seq results has been added to the Results section and we also refer to this data briefly in the Discussion section.

*- “They disprove the hypothesis that Nrs1 competes with the negative regulator (Whi5.) Most of the data supporting a role for Nrs1 in promoting start relies on ectopic over-expression. The nrs1 deletion has no effect on growth rate or cell size in any medium including nitrogen limitation. There are no tested conditions in which nrs1- delays Start or affects fitness.”*

As correctly pointed out by the reviewer, the apparent absence of an obvious cell cycle or growth phenotype for the *nrs1∆* strain was puzzling. In the original submission, all of the experiments reported were performed in various auxotrophic derivatives of the S288C background (BY4741). To allow strain growth media were supplemented with the required amino acids to complement these auxotrophies. It is therefore possible that these additional sources of nitrogen may have masked potential growth phenotypes of *nrs1∆*. In addition, it is possible that laboratory strains may have evolved a reduced requirement for *NRS1*. To circumvent these potential caveats, we deleted *NRS1* in a wild yeast strain, *Saccharomyces boulardii*, which shares >99% of its genome with *S. cerevisiae* (including *NRS1*), and compared the growth of wild type and *nrs1Δ* *S. boulardii* strains. These experiments revealed that cells lacking *NRS1* grow slower than wild type cells in nitrogen-poor medium (YNB, not supplemented with amino acids) but not in nitrogen-rich medium (SC). These results on *nrs1∆* growth defects have been added to the revised manuscript as Supp. Figure S2G, and a new paragraph in the Results section.

A second but not mutually exclusive possibility we considered is that the absence of fitness defects for the *nrs1∆* mutant might be due to genetic buffering in the Start machinery. Because *NRS1* appears to act via SBF-dependent transcription, we hypothesized that effects of *nrs1* deletion might be more evident in the absence of MBF function. To test this idea, we examined growth epistasis between *mbp1∆* and *nrs1∆* mutations. While both deletions and the double deletion had minimal effects on growth in SC-based medium, the *mbp1∆ nrs1∆* double mutant grew substantially slower than the *mbp1∆* mutant in nitrogen-limited YNB-based medium. Thus, in absence of MBF, *nrs1∆* is detrimental to growth when nitrogen is limiting. These data are provided in Supp. Figure S2F and described in the Results section.

*- “The over-expressed GAL:NRS1 is smaller than wild type, suggesting an acceleration of the G1 to S transition, and this size shift requires Swi4 and Swi6. GAL:NRS1 is also smaller than cln3 mutants. The GAL:NRS1 rescue of swi4ts mbp1 (no activators present) is very slight …“*

We agree with the reviewer that the rescue by *NRS1* over-expression as originally shown was indeed somewhat subtle. To further convince the reviewer of this rescue, we have added raw images of the same plates that are shown in Figure 5B of the original submission (now Figure 6B), but taken 2 days later, as a new panel (Supp. Figure 4D). The improved growth of *GAL1-NSR1* *swi4-ts mbp1Δ* strains (for 4 different clones) relative to *swi4-ts mbp1Δ* parental strain is more evident after 2 additional days of growth. However, due to the slow growth rate of the *GAL1-NSR1* *swi4-ts mbp1Δ* compared to wild type and weaker mutants, after 2 additional days of growth, the faster growing strains become saturated, obscuring the growth differences for the control strains. Hence, we decided to keep the original version of Figure 5B (now Figure 6B) in the revised version, and refer to the additional data in Supp. Figure S5D.

*- “ … but the rescue of SWI6SA4 GAL:WHI512A (which leaves the repressive Whi5 on the SBF complex) is quite convincing. Overall, Nrs1 is an interesting small (108aa) protein that directly associates with key Start-regulators for which there is no tangible role at this time. Their model is that Nrs1 bypasses the normal Start controls under nitrogen limitation—but why would cells do that? “*

We agree with the reviewer that this is an interesting question from the perspective of species evolution. We cannot provide a definitive hypothesis to test at this moment because it is largely unknown how the canonical Start machinery is affected in wild type cells by poor nitrogen conditions. We speculate that the acquisition of *NRS1* represents an evolutionarily facile mechanism to confer additional responsiveness to nutrient stresses and optimize the balance between growth and division in nitrogen-limited environments. As described above, as we have now identified circumstances where *NRS1* does contribute to cellular fitness, we suggest that *NRS1* evolved to buffer the Start machinery against severe nitrogen limitation.

*- “The work is of high quality and a lot of effort has been made, but their conclusions rely heavily on overproduction phenotypes. Two resources that would help firm these conclusions would be flow cytometry to show that the G1 to S transition is affected by Nrs1, and transcript measurements demonstrating its activatory role at the anticipated target genes. “*

As described above, genome-wide transcriptional profiles have demonstrated the ability of *NRS1* to activate the Start program. While we have performed flow cytometry experiments to estimate how *NRS1* overexpression affects cell cycle distribution at the cell population level, we did not observe an obvious difference between wild type and *GAL1-NRS1* strains. Similarly, using Whi5 nuclear exit as a proxy for Start activation, we did not observe any obvious difference in homeostatic populations of pre-Start cells grown overnight. In addition, given the newly discovered effects of the *nrs1* deletion on cell growth rates, it is possible that *NRS1* also directly influences cell growth, thereby complicating interpretation of cell cycle distribution data (e.g., activation of Start could be partially masked by increased growth rate). We note that a master regulator of ribosome biogenesis, Sfp1, has recently been found influence the expression of the G1/S regulon by the Shore laboratory. The intricate relationship between Start activation and growth is an interesting and under-appreciated aspect of size control but we hope that the reviewer will agree that further investigation of Nrs1 function in this regard is reasonably outside the scope of the current study.

*“Other comments to improve the manuscript:*

*1.	The chromatin IPs show two-fold effects. There are no error bars and no information about number of replicates.”*

This experiment has been performed in duplicate. Averaging over the two duplicates now yields a 3-4 fold effect. We have added this information and error bars on the new version of former Figure 4C (now Figure 5A) in the revised version of the manuscript.

*“2.	Rescue of swi4ts mbp1 by GAL:NRS1 is almost invisible in Figure 5B and can't be seen in Fig S4B. Can you get a better rescue at a lower temperature or with just swi4ts at a higher temperature? What do C2, C7 etc refer to?”*

C2, C7, C11, C12 refer to four independent *GAL1-NSR1* *swi4-ts mbp1Δ* transformants tested in this assay. To clarify, we have renumbered these four clones C1, C2, C3 and C4 in the revised version of the manuscript. As explained above, the rescue was more apparent after 2 more days of growth (Supp. Figure S4D).

*“3.	The size differences in Fig 6C are very small and reflect a broadening of size distribution more than a significant difference.”*

The purpose of Figure 6C (now Figure 7C) was to demonstrate that fusing Nrs1 to Whi5 at a stoichiometrically equal level could activate Start in the absence of strong *NRS1* overexpression. In this context, the *NRS1-WHI5* fusion gene causes a strong small size phenotype on glucose medium, and a small size phenotype nearly equal to *NRS1* overexpression on galactose medium. We have attempted to clarify the reasoning behind this experiment in the revised text.

*“4.	SGD reports that over-expression of Nrs1 slows vegetative growth—is that a strain difference?”*

We thank the reviewer for pointing this out. The SGD annotation comes from Sopko et al. 2006 (“Mapping pathways and phenotypes by systematic gene overexpression" Mol Cell 21:319-30). In this article, growth was quantified using colony size on plates compared to wild type. Close inspection of Figure 5B of the original submission (now Figure 6B) shows slightly smaller colonies for *GAL1-NRS1* compared to wild type cells in galactose medium. We have not emphasized this since the colony size phenotype is subtle but can include mention of this in the manuscript if the reviewer believes this would be warranted. In addition, the Sopko et al. Mol. Cell 2006 article also lists Swi4, Swi6 and Mbp1 within a set of toxic genes whose overexpression slows down vegetative growth.

*“5.	You didn't get BCK2 or CLN2 in your initial high copy screen. Do they rescue?”*

*BCK2* and *CLN2* did not score in the original screen. We did not investigate this further but it is possible that these gene fusions to GST in the library did not have full function, that over-expression of each gene alone causes growth phenotypes that mask any potential rescue, and/or that the rescue is not strong enough to overcome *WHI5* overexpression in the context of the screen.

**Response to Reviewer #2:**

*- “The paper by Tollis et al reports a new transcriptional activator (Nrs1), which activates G1/S transcription in budding yeast under nitrogen limitation. The manuscript is a revised version. This reviewer did not see the original manuscript. “*

We apologize for the confusion, but the manuscript is an initial submission. The online submission occurred in two steps due to missing information in the first version.

*- “Overall, the discovery of a new regulator of G1/S transcription, especially one that appears to be sensitive to nutrient limitations, would be a significant finding, in this heavily studied field. The authors did an over-expression screen, and found that Nrs1 in high dosage (its expression induced by galactose in this case) enabled cells that lacked other regulators to go through the G1/S transition. It also strongly reduced cell size, consistent with its role as an activator of Start. A variety of follow-up work is supportive of such a role. Overall, the work is well done and I have no issues with technical aspects. My main concern is that of significance and physiological relevance. All the results deal with over-expression of Nrs1. Apparently, deletion of Nrs1 has no effect under any nutrient conditions (including when it is normally expressed, in poor nitrogen sources). What we are left with is a phenotype evident only when a poorly conserved short protein is over-expressed. Furthermore, in the vast majority of the assays in the figures, the experiments were done in media in which Nrs1 is not even normally present (because Nrs1 is normally around only under nutrient limitation). Can one then confidently conclude that just because ectopic expression of Nrs1 can do things at G1/S, then Nrs1 normally does the same things in wild type cells? “*

The reviewer raises a key concern about the physiological role of *NRS1*, which we recognize is an issue. Although some experiments were performed in the context of *NRS1* overexpression, others were in the context of expression from the endogenous locus. In particular, *NRS1* expressed from its endogenous promoter binds to SBF and can be detected on promoter DNA of SBF-regulated genes (Figure 4 of initial submission, either upon rapamycin treatment or in nitrogen-limited media). We also quantify the level of endogenous Nrs1 protein under physiological conditions and find that it approximately stoichiometric with SBF (Figure 2C). Nonetheless, we agree that the absence of an obvious strong phenotype for *nrs1Δ* mutant was a shortfall of the original manuscript. We now report two new results to address this issue:

First, since *NRS1* acts via SBF-dependent transcription, we reasoned the phenotype of the *nrs1Δ* mutant might be revealed when MBF activity is compromised, i.e., such that the cell relies entirely on SBF. We therefore examined growth epistasis of *mbp1∆* and *nrs1∆* deletions. While both deletions and the double deletion had minimal effects on growth in nitrogen-replete SC medium, the *mbp1∆ nrs1∆* double mutant grew much slower than either single mutant in nitrogen-limited YNB-based medium. Thus, in absence of MBF, *nrs1∆* is detrimental to growth under conditions of nitrogen limitation. This result is consistent with other aspects of *NRS1* regulation and activity. We note that like *NRS1*, the loss *MBP1* causes negligible phenotypes in rich nutrient conditions but its role is revealed when SBF activity is compromised.

Second, we considered the possibility that laboratory strains may have lost some of the requirement for NRS1 in the course of decades-long artificial selection. We also note that our original experiments were performed in an auxotrophic strain S288C background (BY4741), such that the amino acids required to complement BY4741 auxotrophies may have served as a sufficient nitrogen source to mask the role of NRS1. To test this possibility, we deleted *NRS1* in a wild fully prototrophic yeast strain, *S. boulardii*, that shares >99% of its genome with *S. cerevisiae* (including NRS1), and compared the growth of wild type versus *nrs1Δ* *S. boulardii* cells. These experiments revealed that cells lacking *NRS1* grow slower than wild type cells in nitrogen-poor medium (YNB, not supplemented with amino acids) but not in nitrogen-rich SC medium.

Both of these new experiments demonstrate the role of *NRS1* in promoting growth in conditions of nitrogen limitation. This data has been added to the revised manuscript as new panels to Supp. Figure 2F and G, and a new paragraph has been added to describe the *nrs1∆* growth defects in the Results section.

**Response to Reviewer #3:**

*- “In this interesting manuscript Tollis et al. describe Nrs1, a new budding yeast cell cycle regulator that promotes passage through Start in G1 under nitrogen limiting conditions. NRS1 (Nitrogen-Responsive Start Regulator 1) encodes a small nuclear protein that it is only expressed when TORC1 is downregulated, either by Rapamycin treatment or by growing the yeasts cells in nitrogen-poor media. Nrs1 interacts genetically and physically with SWI4 and SWI6, which encode components of the Start transcription factor SBF. Consistent with all of these findings, overexpression of NRS1 causes a small cell size phenotype. Most experiments in this manuscript are well executed and the data is very clear. However, there are some experimental details and comments that need to be addressed before accepting this manuscript for publication in PLOS Biology.”*

*“Experimental detail:*

*Table S2 and page 13. Are the strains used in this study prototrophic? This is an important point because the supplements (histidine, leucine, methionine, uracil,…) that have to be added to the minimal medium (YNB) are nitrogen sources that activate TORC1 (Stracka 2014, JBC 289: 25010-25010).*

*As described on page 13 (last paragraph), the nitrogen-limited media used in this manuscript contains supplements: "... nitrogen-limited (0.17% YNB, 0.4% proline, supplemented with histidine, leucine, methionine and uracil to complement auxotrophies as needed)". This point has to be clarified and key experiments should be repeated using prototrophic strains. “*

We thank the reviewer for requesting clarification of these details. Our work was performed in an auxotrophic strain background (BY4741), and hence media were supplemented with the required amino acids to complement BY4741 auxotrophies. As noted in our response to Reviewers #1 and #2 above, this may explain why we did not observe growth or size phenotypes for the *nrs1* deletion mutant in the original submission. With respect to key experiments, we do not believe there is any advantage to repeating experiments with over-expressed *NRS1* in a prototrophic strain because over-expression alone was sufficient to elicit size phenotypes and genetic interactions. Similarly, the fusion of Nrs1 to Whi5 was expressed from the *WHI5* promoter, which is not overtly nutrient regulated. and these experiments were also performed in nitrogen-replete medium. The experiments performed with rapamycin are also immune to nutrient effects because rapamycin dominantly inhibits the mTORC1 complex. As described above, however, we did re-investigate the role of *NRS1* in a fully prototrophic wild type *S. boulardii* strain background, which allowed us to demonstrate a growth phenotype in the absence of rich exogenous nitrogen sources.

*“Major points:*

*1.	If Nrs1 is a positive regulator of START in nitrogen-poor media, then nrs1∆ cells should show a delay in G1. This could be tested using prototrophic strains in YNB-proline without supplements and in other nitrogen-poor media, such as YNB-phenylalanine or YNB-isoleucine.”*

We agree with the reviewer's comment and prediction. As described above, we compared the growth of prototrophic *Saccharomyces boulardii* strains (i.e., wild type and *nrs1Δ*) in nitrogen-rich medium (SC) and nitrogen-poor medium (YNB+Pro without supplements). Cells lacking *NRS1* grew slower than wild type cells in nitrogen-poor medium specifically (new Supp. Figure S2G), uncovering a role for NRS1 as a nitrogen-dependent growth activator. While we did examine DNA content by FACS in this experiment, we did not observe an obvious G1 delay. We suspect the cell cycle phenotype is too subtle to detect by FACS, which is not a particularly sensitive measure of G1 progression. Furthermore, our genome-wide analysis of the transcriptional response to *NRS1* induction (new Figure 5C, Supp Table 6) also identified several ribosomal proteins and other regulators of cell growth downstream of *NRS1*. Hence, it is possible that in addition to activation of the G1/S regulon *NRS1* also directly promotes cell growth, thereby complicating interpretation of cell cycle distribution data (e.g., premature Start activation could be partially masked by increased growth rate). The intricate relationship between Start activation and growth is an interesting and under-appreciated aspect of size control but we hope that the reviewer will agree that further investigation of Nrs1 function in this regard is reasonably outside the scope of the current study.

*“2.	Does Nrs1 inhibit the interaction of Whi5 to the SBF promoters in nitrogen-poor media without supplements?”*

In the original submission, we demonstrated that *GAL1-NRS1* overexpression affected neither the Whi5 interactions with SBF (Supp. Figure 3C of original submission, now Supp. Figure S4C) nor Whi5 binding to SBF-regulated promoters (Supp. Figure 3A, now Supp. Figure 4A). In *GAL1-NRS1* cells, Nrs1 levels are in the micromolar range throughout the cell, i.e. >10-fold higher than endogenous Nrs1 expression in nitrogen-limited media or upon rapamycin treatment (Figure 2), but also >5-7 fold higher than Whi5 protein expression in N-limited medium (Dorsey et al. 2018 and our unpublished data). Since even high level *NRS1* expression does not alter Whi5 interactions, yet does induce G1/S transcription, we do not think that Nrs1 acts by outcompeting Whi5. We examined this possibility previously using in vitro binding experiments with recombinant proteins, now shown in Figure 4D. Increasing amounts of His6-tagged Nrs1 (0.1 ug, 0.2ug and 0.5ug) were incubated with ^FLAG^Swi4-Swi6-^GST^Whi5 complexes (0.1 ug of GST-Whi5, corresponding to a Nrs1:Whi5 molar ratio of about 4, 8 and 20 respectively), immunoprecipitated using anti-FLAG beads to capture SBF and probed for GST to detect Whi5. Co-immunoprecipitation with an irrelevant recombinant protein complex (Cdc4-^FLAG^Skp1) on anti-FLAG beads served as a control for specificity. These experiments show that the interaction of Whi5 with the Swi4-Swi6 complex is not affected by the presence of excess Nrs1. Taken together, this in vitro data in conjunction with the *in vivo* data strongly suggest that Nrs1 does not outcompete Whi5 for SBF binding.

*“3.	Page 10, last paragraph. The authors construct a strain expressing the fusion protein Whi5:Nrs1:GFP from the endogenous WHI5 promoter. These cells showed a reduced cell size similar to whi5∆ cells. One possible explanation for this phenotype and other phenotypes described in the manuscript could be that Whi5:Nrs1:GFP is a non-functional protein and behaves like the WHI5 deletion.”*

We agree with the reviewer that non-functional fusion proteins are always a possibility, especially when combining proteins that have countervailing functions as for Nrs1 and Whi5 in this instance. However, in our original submission, we attempted to control for this possibility. We thus showed that the Whi5-Nrs1-GFP fusion protein is not cleaved (Supp Figure S5A of original submission, now Supp Figure S6A), that it is expressed at levels close to wild type Whi5 levels (Figure 6A, now Figure 7A), that its localization is cell cycle regulated identically to wild type Whi5 (Fiure. 6A, now Figure7A) and that its small size phenotype relative to wild-type in standard SC+2% glucose medium is not as severe as for a *whi5Δ* strain (Figure 6B, now Figure 7B). These results indicate that the fusion protein preserves at least some of the Whi5 inhibitory function and regulation. As a further control to ensure that the Nrs1 fusion did not merely inactivate Whi5 in a non-specific manner, we sought to fuse to Whi5 an inactive mutant of Nrs1. We took advantage of the fact that the evolutionary conserved C-terminus of Nrs1 is transcriptionally inactive (Figure 5A of the original submission, now Figure 6A) and fused it to Whi5 (i.e., a *WHI5-NRS1^Cter^-GFP* strain). Unlike cells expressing the *WHI5-NRS1-GFP* fusion protein, cells expressing *WHI5-NRS1^Cter^-GFP* were not smaller than wild type, and did not rescue *cln3Δ bck2Δ* lethality (new Supp. Figure S6). Overall, these data suggest that Whi5 retains function in the context of Whi5-Nrs1-GFP construct but inhibits SBF less efficiently due to the constitutive presence of full length Nrs1 in the fusion protein.

*“Other comments:*

*1.	In most western blots (Figs. 2B, 4A, S1E), the authors immunoprecipitate the proteins before doing the immunoblot. Is this necessary because Nrs1, Whi5, Swi4 and Swi6 protein levels of are very low? This should be mentioned in the text.”*

As correctly pointed out by the reviewer, Nrs1 (this study) and Whi5, Swi4 and Swi6 (Dorsey et al. Cell Systems 2018) have low expression levels (60-150nM range). This is the reason why we immunoprecipitated the proteins before doing the immunoblots. We have specified this point in the revised version of the Methods.

*“2.	Genetic interactions:*

**	Are the swi4∆ nrs1∆, swi4ts nrs1∆ and swi6∆ nrs1∆ double mutants larger than the swi4∆ or swi6∆ single mutants?*

**	… and mbp1∆ nrs1∆ and mbp1∆ OPnrs1?”*

These are all good suggestions to substantiate a role for Nrs1 at Start. Epistasis of *nrs1∆* with *swi4∆*, *swi4ts* or *swi6∆* could have been used to demonstrate that Nrs1 acts through SBF, provided we had a detectable size phenotype for the *nrs1∆* deletion, which unfortunately was not the case. In the original submission, we showed that Nrs1 acts through SBF by size epistasis of *GAL1-NRS1* with *swi4∆*, *swi4ts* and *swi6∆*. However, prompted by the reviewer's suggestion, we realized that we could exploit the redundancy between SBF and MBF to test the predicted role of *NRS1* in augmenting SBF function when MBF is absent, particularly because we had already shown that Nrs1 does not appear to interact physically with MBF (Figure 4 of the original submission). We examined the growth epistasis of *mbp1∆* and *nrs1∆* mutations and found that while both deletions and the double deletion had no effect in SC-based medium, the *mbp1∆ nrs1∆* double mutant grew considerably slower than the *mbp1∆* mutant in nitrogen-limited YNB medium. Thus, in absence of MBF, the *nrs1∆* mutation is detrimental to growth. Importantly, this genetic interaction also provides further support for the role of *NRS1* in Start activation. These data have been added to the revised manuscript as a new Supp. Figure S2F and a new paragraph describing these data has been added to the Results section.

*“3.	Page 10, Figure 5B. The rescue of swi4-ts mbp1∆ by GAL1-NRS1 is very weak. Could this be tested by PCR?”*

The rescue effect is indeed subtle as the reviewer points out, although it is clearly evident and reproducible. To substantiate the rescue phenotype, we have added images of the same plates shown in Figure 5B of the original submission (now Figure 6B) but taken 2 days later. This data is shown in new Supp. Figure 4D. Specifically, the improved growth of the *GAL1-NSR1* *swi4-ts mbp1Δ* strain (4 different clones, columns 6-9 below starting from left) relative to *swi4-ts mbp1Δ* (column 5) in galactose is more evident after 2 further days of growth. However, due to the slow growth rate of the *GAL1-NSR1* *swi4-ts mbp1Δ* strains compared to wild type the faster growing strains are overgrown after the longer incubation time so we decided to keep the initial version the figure and provide the additional timepoint as a supplementary figure.

*“4.	Fig. S4B, why swi4ts cells grow worst then swi4ts mbp1∆?”*

This is a puzzling observation that we do not have an immediate explanation for other than to say the degree of overlap in SBF/MBF function at Start, and the interplay between Swi4 and Mbp1 in DNA/Swi6 binding and transcription activation is not fully understood. The *swi4-ts* mutant encodes a C-terminally truncated version of Swi4 (Swi4ΔCter) that is impaired in Swi6 binding. As Swi4 and Mbp1 are known to compete for Swi6 it is possible that residual Swi4-Swi6 association at the semi-permissive temperature allows SBF function in absence of the competitor Mbp1, but not in its presence (i.e., Mbp1 might outcompete the defective Swi4ΔCter for Swi6 binding). In any case, at 23^o^C, *swi4-ts mbp1Δ* and *GAL1-NSR1* *swi4-ts mbp1Δ* grow equally well, which is the appropriate control for the experiment.

*“5.	Fig. S1B. After Rapamycin treatment of the Nrs1-Myc tagged strain, the anti-Myc antibody detects two bands. Is the upper band a phosphorylated version or Nrs1? Is Nrs1 regulated by protein phosphorylation?”*

To address this question, we treated Nrs1^MYC^ immunoprecipitates from a rapamycin treatment time course with lambda phosphatase, in the absence (P) or presence (P+I) of phosphatase inhibitors, prior to detection by immunoblot. Phosphorylated Sic1 was used as a control to demonstrate activity of the phosphatase. This experiment showed that that upon rapamycin treatment cells first accumulate a slower-migrating form of Nsr1 that mostly converts to a fast-migrating form but that the slower (presumably modified) form is not sensitive to the powerful but non-specific lambda phosphatase. This result suggests that the apparent post-translational modification of Nrs1 is not due to phosphorylation. The mass spectrometry profiles of the Nrs1 peptides detected in our proteomics experiments (performed on endogenously expressed Nrs1, Table 4) did not reveal any phosphorylated peptides on Nrs1. Although we cannot entirely rule out phosphorylation-dependent regulation of Nrs1, these results suggest the altered form of Nrs1 is not due to phosphorylation. We have added this phosphatase treatment experiment as new Figure 4B and added a short new paragraph to the Results section.

*“6.	Fig. S3C. Are there two bands of Whi5-HA?”*

Yes, there are two species of Whi5-HA in Supp. Figure 3C of the original submission (now Supp. Figure 4C). These species were reported in the original biochemical characterization of Whi5 (Costanzo et al 2004) but the identity of the presumptive modification remains unknown. We have tried to identify the corresponding post-translational modification using mass spectrometry and the modified form is not affected by phosphatase treatment.

*“Minor points:*

*Page 3, Introduction, 2nd paragraph: "spindle body"should be "spindle pole body".*

*Page 6, 1st paragraph: "Figure S1D" should be "Figure S1D and S1E". Figure S1E is not mentioned in the manuscript.*

*Figure 5B: use the same panel arrangement as in Figure S4B (glucose, raffinose and galactose).*

*Figure 5C: indicate that the left panels correspond to Glucose and the right panels to Galactose.*

*Figure legend to Figure S3B: "6 h induction", remove "induction".*

*Figure legend to Figure S4A: with "plasmids" expressing.”*

We thank the reviewer for noticing these errors, which have been corrected in the revised version of the manuscript.

**Response to Reviewer #4:**

*“In this manuscript, Tollis, Singh et al. identify a recently evolved microprotein of budding yeast, Ylr053c/Nrs1, as a dosage suppressor of G1 arrest of cln3∆ bck2∆ double mutant cells, and investigate its contribution to size control at START in nitrogen-limited medium.*

*In budding yeast cell size is controlled in a late G1 cell cycle commitment point called START. When the adequate cell size is reached, cells activate the G1/S transcriptional program by antagonizing the Whi5 inhibitor of SBF-driven transcription and by activating the G1-CDK positive feedback loop that pushes cells towards S phase. The most upstream G1 cyclin Cln3 and a poorly characterized Bck2 together contribute to the initial inactivation of Whi5. Strikingly, cell size at START is modulated by growth conditions, with cells being larger in nutrient-rich medium than in nutrient-limited medium.*

*How nutrients contribute to Start control has been studied for decades, but some players still seem to be missing. Indeed, while cln3∆ bck2∆ cells are unable to pass Start, deletion of WHI5 bypasses this arrest. Strikingly, triple mutant cells maintain nutrient-dependent size control, suggesting that another pathway contributes to size control in the absence of Whi5.*

*In order to identify new players involved in this mechanism, Tollis et al. conducted an elegant yeast overexpression screen looking for dominant suppressors of cln3 bck2 G1 arrest. Doing so, they identified a poorly characterized ORF, which they named NRS1. This new protein falls in the category of microproteins, which are defined as rapidly evolving proteins that give plasticity to cells to adapt quickly to changing environment. The authors use a wide range of well-designed and beautifully-performed yeast genetic experiments, quantitative microscopy, and in vivo or in vitro biochemical approaches to propose a molecular model in which Nrs1 counteracts the Whi5 inhibitory role to promote SBF gene transcription and START in nitrogen-limited conditions. This conclusion is substantiated by showing that i) Nrs1 is strongly induced in nitrogen-limited media or upon TORC1 inhibition by rapamycin; ii) GAL1-NRS1 lowers the cell size of WT, cln3∆ and bck2∆ cells, but not of whi5∆, swi4-ts or swi6∆ cells; iii) Nrs1 interacts physically with Whi5, Swi4 and Swi6; iv) Nrs1 promotes transactivation of SBF-driven genes by counteracting the repression by Whi5.*

*While there is enough novelty to consider this manuscript for publication in PLoS Biology, it is noteworthy to mention that the authors failed to identify a pathway acting in parallel to the Cln3-Bck2-Whi5 module as initially sought, and that effects on cell size are seen only upon Nrs1 overexpression, not loss of function in any of the conditions they tested. This is honestly acknowledged by the authors. It thus appears that Nrs1's presence boosts SBF transcription by binding and counteracting Whi5 inhibition in nitrogen-limited media, but that its absence does not prevent Start to occur at the right size. “*

We thank the reviewer for these well considered thoughts. We acknowledge that while we did not identify the hoped-for parallel Start activators, we did identify a new upstream branch that converges on the core transcriptional machinery in a nutrient specific-fashion. Although we did not completely anticipate this genetic structure in the original screen we nonetheless believe the screen was successful in identifying a new regulator of the well-studied Start machinery. The design of the screen using the *cln3Δ bckΔ GAL1-WHI5* parental background clearly biased the discovery of new Start activators towards genes able to operate in presence of high *WHI5* dosage, i.e., as obtained for *NRS1*. We have commented briefly on this point in the revised version of the manuscript.

As correctly pointed out by the Reviewer, in the original submission we were unable to document any obvious fitness defect for the *nrs1* deletion mutant. As noted above though, two important new results mitigate this concern. First, our new genetic epistasis experiments with *mbp1∆* and *nrs1∆* have uncovered a role for *NRS1* specifically under poor nitrogen conditions when the buffering effect of MBF is removed. Second, after extensive evaluation different strain backgrounds, we identified a wild strain *(S. boulardii*, which is very closely related to *S. cerevisiae*) that requires *NRS1* function for optimal growth only under poor nitrogen conditions, consistent with the observed *NRS1* regulation in *S. cerevisiae*. Both experiments have been added to the revised manuscript as new Supp. Figure 2F and 2G, and discussed in the Results section.

*“The following points deserve to be addressed before publication:*

*Major points:*

*1.	Does GAL-NRS1 also suppress the G1 arrest of cln1 cln2 cln3 triple mutant? This may give insights to the role of Bck2.”*

We thank the reviewer for this suggestion that further probes the genetic role of *NRS1* at Start. We used a *cln1Δcln2Δcln3Δ MET-CLN2* strain transformed with a *GAL1-NRS1* plasmid to address this question. In the absence of methionine, the *cln1Δcln2Δcln3Δ MET-CLN2* strain grew well, albeit slower than a wild type strain, as expected. Induction of *NRS1* in the absence of methionine made the conditional cln^-^ strain smaller, as it does for wild type, indicating no specific requirement for *CLN1* or *CLN3* in the small size phenotype caused by *GAL1-NRS1*. In the presence of methionine, *cln1Δcln2Δcln3Δ MET-CLN2* cells were unable to grow but this growth defect was not rescued by *NRS1* induction. These data are shown in the new Supp. Figure 5B and mentioned in the Results section. We confirmed this result with a *cln1Δcln2Δ GAL-CLN3* strain using a *TDH1-NRS1* construct to express NRS1 on glucose medium, which also failed to rescue the cln^-^ arrest (data not shown). We conclude that *NRS1* cannot compensate for the complete absence of G1 cyclins and speculate that G1 cyclin-CDK activity is required to activate SBF in addition to Whi5 inactivation, as suggested by previous studies.

*“2.	Nrs1 appears as a doublet on western blots, but as a single band upon TORC1 inhibition by rapamycin. It would be useful to know if this corresponds to a PTM or cleavage of Nrs1. MW markers should be added to estimate the size of this band. Which of the two forms, short or long, does interact with Whi5 and Swi4,5? Also, why are IPs used instead of whole cell extracts? Can Nrs1 not be detected on the latter, why? “*

As correctly remarked by the reviewer, Nrs1 appeared as a doublet in poor-nitrogen conditions but not upon 2 h treatment with rapamycin where only the fast-migrating form was detected (Figure 2B). From the coimmunoprecipitation data shown in Figure 4A it appears that the slower-migrating form of Nrs1 interacts with SBF. We performed immunoprecipitations from a rapamycin treatment time course and found that the slow-migrating form also appeared first upon rapamycin treatment, before conversion to the fast-migrating form after 2 h (new panel Figure 4B). We treated Nrs1^MYC^ immunoprecipitates from the rapamycin treatment time course with lambda phosphatase, in the absence (P) or presence (P+I) of phosphatase inhibitors, prior to detection by immunoblot. Phosphorylated Sic1 was used as a control to demonstrate activity of the phosphatase. This experiment showed that the slow-migrating (presumably modified) form of Nrs1 that interacts with SBF is not sensitive to the powerful but non-specific lambda phosphatase. This result suggests that the apparent post-translational modification of Nrs1 is not due to phosphorylation. The mass spectrometry profiles of the Nrs1 peptides detected in our proteomics experiments (performed on endogenously expressed Nrs1, Table 4) did not reveal any phosphorylated peptides on Nrs1. Although we cannot entirely rule out phosphorylation-dependent regulation of Nrs1, these results suggest the altered form of Nrs1 is not due to phosphorylation. We have added this phosphatase treatment experiment as new Figure 4B and added a short new paragraph to the Results section.

Moreover, we found it was necessary to first concentrate Nrs1 by immunoprecipitation in order to detect the low levels of the endogenously expressed protein (which we estimate at 50-80 nM, Figure 2C). We have noted this point about Nrs1 detection in the revised manuscript.

*“3.	The cell cycle regulation of Nrs1 abundance, its dynamics of induction after nitrogen limitation, and nuclear localization should be better documented. It is expected that a regulator of Start would peak just before the G1/S transition, which could be shown by western blots of synchronized or elutriated cells. It is not so clear from the images provided that Nrs1 is nuclear in late G1 unbudded cells. A few examples of higher resolution images and quantification of the fraction of G1 cells with nuclear Nrs1 would be useful.”*

In the original submission, we showed that Nrs1 induction upon rapamycin treatment is detectable within 30 minutes (Supp. Figure 1B). However, upon nitrogen limitation, cells might have first to deplete nitrogen reserves before *NRS1* expression is induced. Given the difficulty in detecting endogenous Nrs1 described above and the complexities of synchronization and slow growth in poor nitrogen sources, we chose to investigate the issue of cell cycle regulation by single cell imaging methods. To address the point of induction by nitrogen limitation, we repeated the quantitative imaging of Nrs1-GFP cells in YNB+0.4%Pro+2%glucose (supplemented with His, Leu, Met, Ura; Figure 2A,C), but using two different induction times: cells induced for 22h from a 1:5000 dilution of a saturated SC+2%glucose culture into low nitrogen medium and cells induced for 7h from a 1:100 dilution. At least 100 cells were quantitatively imaged in each condition. Nrs1-GFP cells induced for 22h (but not for 7h) showed detectable GFP signal, as now shown in the new Supp. Figure 1B. Hence, unlike for TORC1 inhibition, the induction of Nrs1 by nitrogen scarcity requires several hours. We have briefly discussed these results in the revised version of the Results section.

With respect to the question of cell cycle dynamics of Nrs1 expression, in the original submission, cell cycle regulation of Nrs1 was inferred from low-throughput, high-resolution N&B microscopy experiments that provided an accurate measurement of the peak Nrs1 concentration at G1/S, based on dozens of cells (i.e., Figure 2A,C). To establish more robust statistics, we imaged the same Nrs1-GFPmut3 cells originally imaged by N&B on a high-content Opera confocal platform (cells were grown overnight on nitrogen-limited YNB+pro+2%glu medium as described above). Cells from 18 different fields of view (FOV) were classified into 3 cell cycle phase groups based on morphology (small unbudded; large unbudded and small-to-medium budded; medium-to-large budded), and for each cell the GFP signal was manually scored (strong nuclear = green arrows; dim nuclear = blue arrows; non-nuclear = red arrows, see sample image in the new Figure 2D). Dead cells (white arrows) were not scored nor were out-of-focus cells for which the budding pattern could not be ascertained, and nor were the parts of the FOVs where illumination was not homogeneous. With this manual scoring method, we found that 41% of small unbudded cells had no nuclear Nrs1, 45% had a dim nuclear signal, 14% had a strong nuclear signal (N=708 cells); 22% of large unbudded+small-to-medium budded cells had no nuclear Nrs1, 30% had a dim nuclear signal, 48% had a strong nuclear signal (N=599); 92% of medium-to-large budded cells had no nuclear Nrs1, 6% had a dim nuclear signal, 2% had a strong nuclear signal (N=186). We note that it is possible that some small buds were mis-classified as unbudded cells with strong Nrs1 signal. These results, summarized in histogram format (Figure 2D), confirmed that the peak in Nrs1 expression and nuclear prominence occurred G1 before and at the time of budding, a hallmark of the G1/S transition. These new results have been included in the revised version of Figure 2D and described in the Results section.

*“4.	It is not known if Nrs1 also boosts MBF-driven transcription. Has this been tested or is it not relevant? A comment would be welcome.”*

This is a good question given that some aspects of SBF and MBF regulation are distinct (e.g., Whi5 mainly inhibits SBF, Nrm1 specifically inhibits MBF). In the original submission Figure 4B (now Figure 4C), we showed that Nrs1 did not interact with MBF in our in vitro experiments and so we did not pursue possible regulation of MBF by Nrs1. We identified some MBF targets genes as upregulated upon *NRS1* induction in the new RNA-seq experiments (see above, new Figure 5C and Table 6); however because of the partial functional overlap between SBF and MBF, it is possible that MBF targets are upregulated via Nrs1-dependent SBF transcription only. Finally, since our new genetic interaction data shows a synthetic growth defect of the *mbp1 nrs1* double mutant (see above), we suspect that Nrs1 and MBF act in parallel pathways to activate Start, consistent with an SBF-specific function for Nrs1. As noted above, we have added text to the revised manuscript on this point.

*“5.	The initial SGA screen identified 12 high-confidence hits. It is shown that YEA4 was not confirmed in a direct test. What about the other hits?”*

*NRS1* and *CLN3* were the only other hits confirmed by direct transformation of the query strain. We have clarified that none of the other hits from the screen validated in the revised version of the manuscript, as above.

*“6.	Size control by low nitrogen or poor carbon sources is used interchangeably in the text. It would be useful to better distinguish the two controls in the introduction and be more precise in figure legends on which conditions have been used, and for how long.”*

We thank the reviewer for raising this point, as it is critical that readers understand the nutrient conditions in our experiments. We have modified the text as necessary to make the nutrient conditions clear for each experiment we describe, in particular by avoiding the use of the term poor nutrients when we mean nitrogen limitation (or poor nitrogen).

*“Minor points:*

*1.	two different concentrations of rapamycin are used in the manuscript. 100 nM for the experiments shown on figures 2 and 200 ng/mL for those on the figure S1, figure 4C. Is there a biological reason for using these 2 different concentrations? Moreover, the length of the rapamycin treatment applied before cell imaging (figure 2A) or protein extracts preparation (figures 2B, S1E, 4A) should be given.”*

For all experiments except the rapamycin treatment time course in Supp. Figure 1B and the sN&B imaging in Figure 2A, cells were grown to log-phase and then treated with 200 ng/mL (about 200nM) rapamycin for 2 h as the default rapamycin concentration. The same concentration was used for the time course (Supp. Figure 1B of the original submission, now Supp. Figure 1C), only the duration of treatment was different, as required by the time course (i.e., from 0 to 3h). To cope with the technical constraints of sN&B experiments, the 200nM rapamycin dose was added in 2 steps: 100nM was added to growth medium 1h before sample preparation, and 100nM at the beginning of sample preparation. Sample preparation lasted about 30 min, and imaging 1-1.5 h, resulting in a total of 2-3 h of rapamycin treatment for the different FOVs. We did not observe substantial differences in Nrs1 levels across FOVs acquired at the beginning and end of imaging sessions. We have clarified the rapamycin treatment protocols in the Methods section of revised version of the manuscript.

*“2.	The meaning of the dN/dS ratio and how it is calculated would help non-specialists of evolutionary biology if details were given in the material and methods or in supplemental methods.”*

We have clarified the definition of the dN/dS ratio in the revised manuscript.

*“3.	Figure S1B: To support the claim that Nrs1 is transiently expressed, a loading control should be added to this figure. Additionally, an input of a better quality would be appreciated.”*

The transient expression of Nrs1 upon rapamycin induction was shown in the original Supp. Figure 1B (now Supp. Figure 1C) both by immunoprecipitation followed immunoblot (which was necessary to reliably detect Nrs1 signal) and by direct immunoblot for this one experiment. Unfortunately, we do not have a loading control for the direct immunoblot. However, this result is backed up by multiple different single cell imaging analyses throughout the manuscript, all of which are consistent with the immunoblot data. In the revised version of the manuscript we have removed reference to transient induction of Nrs1 upon rapamycin treatment since we originally intended for this phrase to refer to the changes in distribution between the slow and fast migrating forms of Nrs1.

*“4.	Figure 4A: To support their model, the authors should show if Nrs1 and Swi4 or Swi6 interact when cells are grown in nitrogen-limited source medium.”*

In the original submission, we performed mass spectrometry-based proteomics on otherwise wild type cells bearing an integrated *NRS1^13MYC^* allele and grown in nitrogen-limited media (Supp. Figure 2F of original submission, now Supp. Figure 3; also Tables 3, 4, 5). Proteins detected in the anti- MYC-immunoprecipitates were identified by subtracting proteins present in the untagged control and filtering hits against the CRAPome database of non-specific interactions. 7 proteins were found specifically it the Nrs1^13MYC^ sample: Nrs1, represented by 7 peptides (48% coverage), and critically both Swi4 and Swi6 were represented by 5 peptide each. This unbiased analysis demonstrates that endogenous Nsr1 expressed upon nitrogen deprivation also interacts with SBF, just as the endogenous Nrs1 expressed upon TORC1 inhibition does (Figure 4A). We have revised the text to make this point more evident.

*“5.	Figures 4B and S3D: The origin of the recombinant GSTNrs1 fusion protein is not described. Is it expressed from the locus or from a plasmid? The information should be provided in the strain or plasmid table.”*

The recombinant ^GST^Nrs1 was produced by plasmid-driven expression in E. coli and purified by the GST tag. We apologize for this oversight and have added this information to the revised manuscript.

*“6.	Figures 4C and S3A: It is reasonable to think that ChIP-PCR experiments were performed more than once. Error bars should be added.”*

These experiments have been performed in duplicate. We have added this information and error bars to the new version of former Figure 4C (now Figure 5A) and Supp. Figure 3A (now Supp. Figure 4A) in the revised version of the manuscript.

*“7.	Figure S3C: It is not clear why 2 polypeptides that migrate differently are referred to as Whi5HA. Is the faint upper band hyperphosphorylated Whi5?”*

As described above, the nature of the mysterious Whi5 modification first documented by Costanzo et al (2004) remains unknown but it appears not to be due to phosphorylation.

*“8.	Figure 4D: The authors should indicate if they determined the diffusion constant of the total or nuclear Swi6. If they determine the latter, they should do the same for Nrs1.”*

We apologize for the lack of clarity in our presentation of the diffusion data. With the imaging parameters described in the Methods, each field of view (FOV) typically contained only one or two cells. The diffusion constants shown on Figure 4D of the original submission (now Figure 5B) were computed over the entire FOV, hence over the entire cytosol and nucleus. All samples were analyzed the same way, using both the contributions of the strong nuclear and weak cytosolic fluorescence. The “total” and “cyt” labels for Nrs1 were added to distinguish individual cells where Nsr1 was prominently nuclear, and cells where Nsr1 nuclear localization was not detectable, respectively. The purpose of this distinction was to show the decrease in overall (cell-averaged) Nrs1 mobility associated with the localization of the Nsr1 signal in the nucleus, which shows that nuclear Nrs1 is much less mobile than its cytosolic counterpart. We agree with the reviewer that the labels in the original submission were confusing, and we have replaced these by “Nrs1-GFP, cells with nuclear signal” and “Nrs1-GFP, other cells” in the revised version of the manuscript, and improved the clarity of the comparison of these two samples in the main text.

*“9.	Figures S5A: An immunoblot is provided to show that the construct Whi5-Nrs1-GFP had the expected molecular weight. It would more convincing if the molecular weight of the ladder was added. Additionally, the difference of expression between Whi5-GFP and Whi5-Nrs1-GFP is really striking. However, protein extracts are prepared from cells grown in nitrogen-limited medium. It is a condition that is never used with cells expressing this construct. An immunoblot performed with protein extracts prepared from cells grown in SC + Glu 2% would be more appropriate and less misleading.”*

In the revised version of the manuscript, we have provided a new version of the immunoblot in Supp. Figure 5A (now Supp. Figure 6A) including a ladder of Mr markers. As all other experiments with the Whi5-Nrs1 fusion, this experiment was performed with SC+2%glucose-grown cells, and we have corrected the Methods accordingly.

This immunoblot experiment was intended to demonstrate the correct molecular weight of the Whi5-Nrs1-GFP construct, not for Whi5-Nrs1-GFP quantification. Hence, the relative expression levels of Whi5 and the Whi5-Nrs1 construct are best not inferred from this experiment. Rather, the expression of the Whi5-Nrs1-GFP construct was carefully quantified by Number and Brightness microscopy (N&B, Figure 6A of the original submission, now Figure 7A). As all other experiments with the fusion protein (with the exception of the test for cleavage, as correctly noticed by the reviewer), these N&B measurements were performed in in SC+2% glucose medium. We indeed found a decrease in Whi5-Nrs1 levels compared to Whi5 in wild type cells, but only by about 20-25%, which is not as striking as on the non-quantitative blot. In principle, a decrease in Whi5 dosage could explain the small size phenotype of the Whi5-Nrs1 fusion strain. However, several lines of evidence argue against a Whi5 dosage-dependence of cell size, as discussed in the main text of the original submission, and supported by new data with the Whi5-Nrs1^Cter^-GFP fusion protein show in Figure 5A and Supp. Figure S7). Hence, the modest reduction in Whi5 dosage seems unlikely to explain the strong small size phenotype associated with the fusion protein.

*10.	Figures S1E, S4A do not appear to be cited anywhere in the text.*

*11.	The type of media (carbon sources) is missing on the panels of the figure5c.*

*12.	A typo on p11 line 2: Figures 6A; Figure S5B…*

We thank the reviewer for catching these errors, which have been corrected in the revised version of the manuscript.

New references added to manuscript:

[45]: Ogas J, Andrews BJ, Herskowitz I. Transcriptional activation of CLN1, CLN2, and a putative new G1 cyclin (HCS26) by SWI4, a positive regulator of G1-specific transcription. Cell. 1991;66(5):1015-26.

[46]: Khatri I, Tomar R, Ganesan K, Prasad GS, Subramanian S. Complete genome sequence and comparative genomics of the probiotic yeast Saccharomyces boulardii. Sci Rep. 2017;7(1):371.

[73]: Ghazal G, Gagnon J, Jacques PE, Landry JR, Robert F, Elela SA. Yeast RNase III triggers polyadenylation-independent transcription termination. Mol Cell. 2009;36(1):99-109.

[74]: Langmead B, Salzberg SL. Fast gapped-read alignment with Bowtie 2. Nat Methods. 2012;9(4):357-9.

[75]: Bullard JH, Purdom E, Hansen KD, Dudoit S. Evaluation of statistical methods for normalization and differential expression in mRNA-Seq experiments. BMC Bioinformatics. 2010;11:94.
